# ATF2 loss promotes tumor invasion in colorectal cancer cells via upregulation of cancer driver TROP2

**DOI:** 10.1007/s00018-022-04445-5

**Published:** 2022-07-15

**Authors:** Kerstin Huebner, Katharina Erlenbach-Wuensch, Jan Prochazka, Ilir Sheraj, Chuanpit Hampel, Blanka Mrazkova, Tereza Michalcikova, Jolana Tureckova, Veronika Iatsiuk, Anne Weissmann, Fulvia Ferrazzi, Philipp Kunze, Enise Nalli, Elisabeth Sammer, Annemarie Gehring, Marie M. Cheema, Markus Eckstein, Eva-Maria Paap, Agnes Soederberg, Corinna Fischer, Sushmita Paul, Vijayalakshmi Mahadevan, Benardina Ndreshkjana, Melanie A. Meier, Susanne Muehlich, Carol I. Geppert, Susanne Merkel, Robert Grutzmann, Adriana Roehe, Sreeparna Banerjee, Arndt Hartmann, Radislav Sedlacek, Regine Schneider-Stock

**Affiliations:** 1 Experimental Tumor Pathology, Institute of Pathology, University Hospital Erlangen, Friedrich Alexander University Erlangen‐Nürnberg (FAU), Universitätsstraße 22, 91054 Erlangen, Germany; 2 Institute of Pathology, University Hospital Erlangen, Friedrich Alexander University Erlangen‐Nürnberg (FAU), 91054 Erlangen, Germany; 3 Czech Center for Phenogenomics, Institute of Molecular Genetics of the ASCR, v.v.i, 142 20 Prague, Czech Republic; 4 Department of Biological Sciences, Middle East Technical University, 06800 Ankara, Turkey; 5 Department of Nephropathology, Institute of Pathology, University Hospital Erlangen, Friedrich Alexander-University Erlangen-Nürnberg, 91054 Erlangen, Germany; 6 Department of Bioscience and Bioengineering, Indian Institute of Technology Jodhpur, Jodhpur, 342037 India; 7 Institute of Bioinformatics and Applied Biotechnology (IBAB), Bangalore, 560100 India; 8 Department of Chemistry and Pharmacy, Molecular and Clinical Pharmacy, Friedrich Alexander University Erlangen-Nürnberg, 91058 Erlangen, Germany; 9 Department of Surgery, University Hospital Erlangen, Friedrich Alexander University Erlangen-Nürnberg, 91054 Erlangen, Germany; 10 Comprehensive Cancer Center Erlangen-EMN (CCC ER-EMN), University Hospital Erlangen, Friedrich Alexander University Erlangen-Nürnberg, 91054 Erlangen, Germany; 11 Department of Pathology and Legal Medicine, Federal University of Health Sciences of Porto Alegre, Porto Alegre, 90050-170 Brazil

**Keywords:** De-adhesion, Migration, Intratumoral heterogeneity, Liver metastasis, EMT, CAM model

## Abstract

In cancer, the activating transcription factor 2 (ATF2) has pleiotropic functions in cellular responses to growth stimuli, damage, or inflammation. Due to only limited studies, the significance of ATF2 in colorectal cancer (CRC) is not well understood. We report that low ATF2 levels correlated with worse prognosis and tumor aggressiveness in CRC patients. NanoString gene expression and ChIP analysis confirmed trophoblast cell surface antigen 2 (TROP2) as a novel inhibitory ATF2 target gene. This inverse correlation was further observed in primary human tumor tissues. Immunostainings revealed that high intratumoral heterogeneity for ATF2 and TROP2 expression was sustained also in liver metastasis. Mechanistically, our in vitro data of CRISPR/Cas9-generated ATF2 knockout (KO) clones revealed that high TROP2 levels were critical for cell de-adhesion and increased cell migration without triggering EMT. TROP2 was enriched in filopodia and displaced Paxillin from adherens junctions. In vivo imaging, micro-computer tomography, and immunostainings verified that an ATF2^KO^/TROP2^high^ status triggered tumor invasiveness in in vivo mouse and chicken xenograft models. In silico analysis provided direct support that ATF2^low^/TROP2^high^ expression status defined high-risk CRC patients. Finally, our data demonstrate that ATF2 acts as a tumor suppressor by inhibiting the cancer driver TROP2. Therapeutic TROP2 targeting might prevent particularly the first steps in metastasis, i.e., the de-adhesion and invasion of colon cancer cells.

**Supplementary Information:**

The online version contains supplementary material available at 10.1007/s00018-022-04445-5.

## Introduction

Colorectal cancer (CRC) is one of the most commonly diagnosed cancers [1]. Most CRC-related deaths are associated with metastatic progression. Metastasis is a multistep and multifactorial process, starting with the dissemination of tumor cells from the bulk tumor and their local invasion into the surrounding extracellular matrix [2]. The molecular and cellular mechanisms underlying these early steps in metastatic spread of CRC are mostly unknown [3]. Thus, the discovery of molecular markers for the identification of highly invasive tumor cells is urgently needed to investigate novel therapeutic targets.

Intratumoral heterogeneity (ITH) exists and arises among cancer cells within the same tumor as a result of (epi-)genetic changes, environmental differences, and cellular plasticity [4]. It reflects distinct tumor cell populations with specific phenotypic, molecular, and functional characteristics. Consequently, ITH is the leading cause of tumor relapse and chemotherapy resistance [5]. The relevance of ITH became highly recognized following the pioneering work of Guinney et al. in defining consensus molecular subtypes in CRC according to specific gene signatures [6]. Though this transcriptome analysis was based on bulk tumor data, it lacked the ability to capture ITH. Recently, gene signatures of single knockout (KO) cells generated by CRISPR gene editing have changed our molecular understanding of ITH by providing an instrument to characterize the diverse cellular and functional populations in a tumor [7], thereby significantly reducing experimental bias.

Activating transcription factor 2 (ATF2) belongs to the family of bZIP transcription factors and is involved in transcriptional regulation, chromatin remodeling, and DNA damage response [8, 9]. As part of the AP1 transcription factor complex, it forms homo-/heterodimers with other bZIP proteins, preferentially c-JUN, that bind to specific DNA motifs via their conserved leucine zipper regions [9]. ATF2 has a highly divergent character, and can either drive or block tumor progression in a tissue- and stimulus-dependent manner [10–13]. In CRC, ATF2 has been highlighted in a global transcription factor network analysis combining topological and biological features [14]. Moreover, ATF2 motifs are enriched in the non-canonical Wnt target cluster in colon cancer cells [15]. In data from The Cancer Genome Atlas (TCGA), a subgroup of CRC patients with poor prognosis had low *ATF2* gene expression [9]. Thus, ATF2 might be closely linked to tumor invasiveness in CRC; however, the pathway remains unknown. Here, we identified a novel ATF2-dependent mechanism underlying tumor invasiveness in CRC in vitro*, *in vivo, and in silico. We observed that the cancer driver trophoblast cell surface antigen 2 (TROP2) is one of the key players in the ATF2 network, associated with de-adhesion and migration potential of cancer cells. The ATF2^low^/TROP2^high^ expression status could be a suitable marker combination to stratify high-risk CRC patients.

## Materials and methods

### Human CRC cohort

This study was covered by ethic votes of the University Hospital of the Friedrich-Alexander University Erlangen-Nürnberg (24.01.2005, 18.01.2012). Tissue microarrays (TMAs) were constructed as previously described [40, 41]. More details are given in the supplemental Material and Methods section. Detailed information for this patient cohort is given in Supplementary Table 1.

### Cell culture

Cell line details, mycoplasma testing, authentication are given in the supplemental Material and Methods section.

### Generation of stable ATF2 and TROP2 knockout cells

Details on CRISPR/Cas9 technique, transfection and validation are given in the supplemental Material and Methods sections.

### NanoString gene expression analysis

Gene expression analysis was performed using the human nCounter® PanCancer Progression Panel (NanoString Technologies, Seattle, WA, USA) according to the manufacturer’s protocol with 100 ng of total RNA from HCT116, F9, and E5 cells. Details on data processing are given in the supplemental Material and Methods sections.

### Bioinformatics analysis

In silico analysis methods and data sets are given in the supplemental Material and Methods section.

### Chorioallantoic membrane (CAM) assay

The CAM assay was conducted as previously described [29]. More details are given in the supplemental Material and Methods section.

### Detection of disseminating tumor cells by Alu qPCR

The dissemination potential of tumor cells upon ATF2 loss was determined by Alu qPCR in chicken embryonic organs based on the CAM assay as previously described [42]. More details are given in the supplemental Material and Methods section.

### RNA interference

Details on RNA interference-mediated gene silencing are given in the supplemental Material and Methods section.

### Western blot

Cells pellets were collected and lysed, and western blotting was performed as previously described [29, 43]. More details are given in the supplemental Material and Methods section. Primary antibodies are listed in Supplementary Table 8.

### RT-qPCR

Total RNA from cell pellets was extracted using QIAzol® Lysis Reagent (Qiagen) combined with RNeasy Mini Kit (Qiagen) according to the manufacturer’s instructions. Primers are given in Supplementary Table 9. More details are given in the supplemental Material and Methods section.

### Chromatin immunoprecipitation

Chromatin immunoprecipitation (ChIP) was performed using the ChIP-IT High Sensitivity Kit (Active Motif, Carlsbad, CA, USA) according to the manufacturer’s protocol. More details about reagents, controls and data evaluation are given in the supplemental Material and Methods section. Primers are listed in Supplementary Table 10.

### Wound healing migration assay

Cells of HT29 and ATF2-KO clone B5 were transfected with *TROP2*-specific (si) or non-targeting (scr) RNAi for 48 h as described in the methods section “RNA interference”. More details are given in the supplemental Material and Methods section.

### 3D tumor spheroid migration assays

The spheroid migration and invasion assay was performed as previously described [29]. More details are given in the supplemental Material and Methods section.

### Immunofluorescence

Details on immunofluorescent stainings and filopodia quantification are given in the supplemental Material and Methods section.

### Immunohistochemical staining and analysis

Details are given in the supplemental Material and Methods section. Antibodies used are listed in Supplementary Table 11.

### Lentiviral vector preparation and cell transduction for luciferase-labeled cell lines

Details of lentiviral preparation and transduction are given in the supplemental Material and Methods section.

### Subcutaneous murine xenograft model and IHC analysis

Mouse experiments were conducted in accordance with institutional guidelines of the Institute of Molecular Genetics, Czech Academy of Science, and approved under the project license PP63-2018. Thirteen- to fifteen-week-old, male, immuno-deficient mice (NOD.Cg-Prkdc^scid^ Il2rg^tm1Wjl^/SzJ) were purchased from The Jackson Laboratory [44, 45] and housed under specific-pathogen-free conditions with daily 12 h light and 12 h dark cycles. More details are given in the supplemental Material and Methods section.

### Micro-CT imaging

Details of Micro-CT imaging are given in the supplemental Material and Methods section.

### JNK pathway modulation

Next, 2.0 × 10^6^ cells were seeded and treated on the next day with JNK inhibitor SP600125. JNK inhibitor treatment (10 µM, in DMSO, tlrl-sp60; InvivoGen, Toulouse, France) was performed for 24 h. Controls for JNK inhibition were treated with equivalent doses of DMSO.

### Anchorage-independent growth assay

To study anchorage-independent growth, 6-well plates were coated twice with polyhydroxyethylmetacrylate (poly-HEMA; Sigma Aldrich, P3932) (20 mg/ml, in 95% ethanol, sterile-filtered) and dried under flow cabinets at RT. Cells were seeded at low density (2.5 × 10^4^ cells/well) in poly-HEMA-coated wells. Formation of aggregates was documented for up to 96 h by light microscopy (Leica DMi1, Leica Microsystems, Wetzlar, Germany) at 4× magnification. Cell cluster sizes were evaluated in ImageJ software using a self-written macro. After 96 h of anchorage-independent growth, HT29 and ATF2-KO cells were stained with 2 µM calcein (Thermo Fisher, Waltham, MA, USA) for 30 min at 37 °C, 5% CO_2_, and imaged by fluorescent microscopy (Nikon Eclipse Ti-S) at 4× magnification.

## Results

### Low ATF2 expression identifies a high-risk subgroup of patients in CRC

To investigate the role of ATF2 in CRC, we examined its protein expression levels on a tissue microarray (TMA) containing samples from 332 CRC patients using an ATF2 score based on immuno-histochemical (IHC) staining (Supplementary Table 1); a predominantly nuclear expression pattern was observed (Supplementary Fig. 1A).

**Fig. 1:**
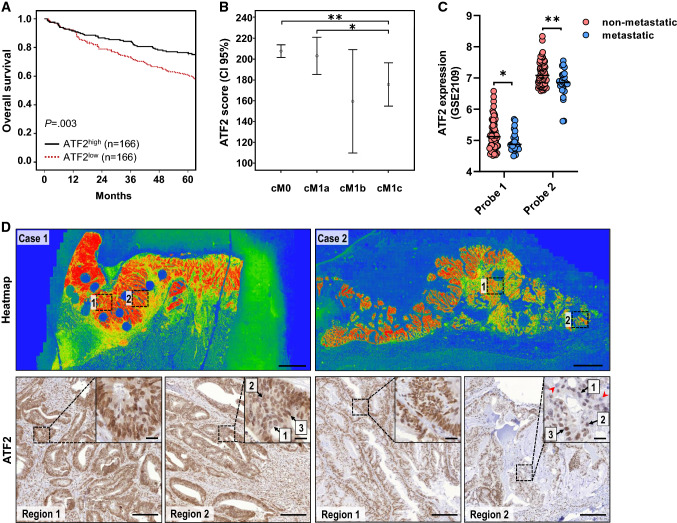
Colorectal cancer (CRC) patients with low ATF2 expression present increased intratumoral heterogeneity and poor prognosis. **A** Kaplan–Meier plot of overall survival in CRC patients (*n* = 332) grouped into high and low according to their median ATF2 protein levels (median = 198, *P* = 0.003, log-rank test). **B** ATF2 IHC score of patients with primary CRC presenting no metastasis (cM0, *n* = 260), solitary organ metastasis (cM1a, *n* = 41), multiple metastasis without (cM1b, *n* = 5) or with (cM1c, *n* = 26) peritoneal involvement (*P < 0.05, **P < 0.01, Mann-Whitney test). **C** *ATF2* RNA expression in metastatic (*n* = 25) versus non-metastatic primary CRC (*n* = 65) as extracted from the GSE2109 dataset. The line shows the median (*P < 0.05, **P < 0.01, Welch’s *t* test). **D** Representative heatmaps and corresponding ATF2 IHC staining of CRC samples (*n* = 20). Heatmap, scale: 2 mm; IHC overview, scale: 200 µm; insert, scale: 20 µm. Color scale: red to green color, high to low staining intensity. Numbers 1–3: staining intensity; holes: positions of TMA punches; red arrowheads: ATF2-negative cells.

Survival analysis revealed that patients with low ATF2 expression had significantly worse overall survival (Fig. 1A). Although univariate Cox regression analysis identified ATF2 and classical clinico-pathological parameters as prognostically relevant, subsequent multivariate Cox regression analysis revealed the presence of only synchronous distant metastasis and lymphatic invasion as independent prognostic markers (Supplementary Table 2). Notably, when the M status for CRC patients was considered unknown, ATF2 could serve as an independent prognostic factor (*P* = 0.018) in multivariate Cox regression analysis. Interestingly, ATF2 expression was lowest in primary tumors that developed multiple metastasis with or without peritoneal involvement at the time of primary diagnosis (Fig. 1B). In silico analysis of gene expression omnibus (GEO) series (GSE) revealed decreased *ATF2* expression in metastatic tumors (Fig. 1C).

Whole tissue sections from our CRC cohort revealed strongly heterogeneous nuclear ATF2 expression levels, with few cell aggregates completely devoid of ATF2 expression (Fig. 1D, Supplementary Fig. 1B). The functional role of this minor ATF2-negative subpopulation is completely unknown.

### ATF2 loss results in increased TROP2 expression

**Fig. 2:**
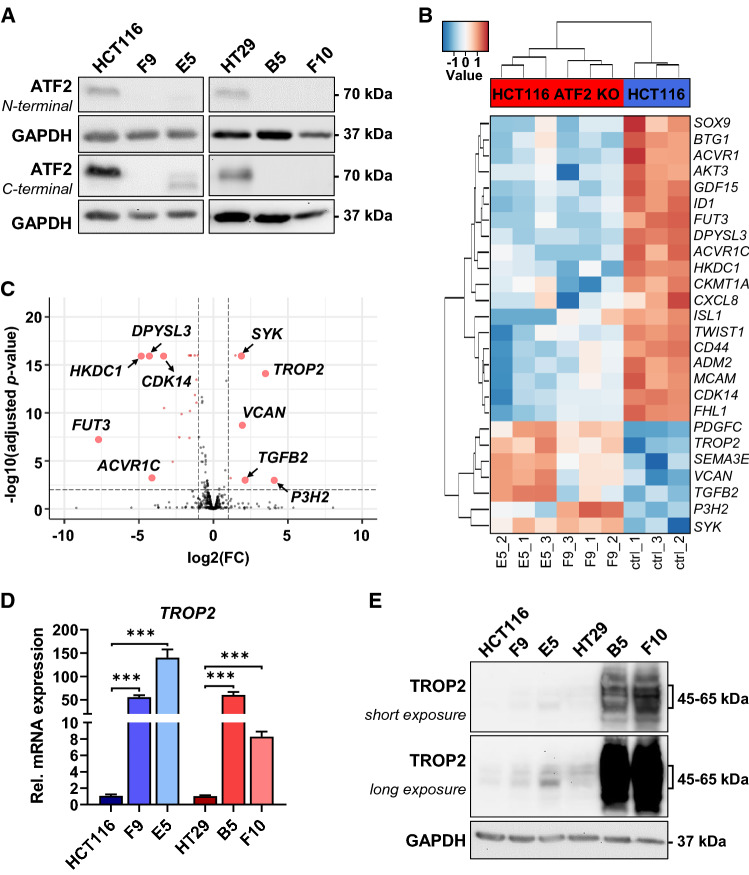
ATF2 loss results in the upregulation of the metastasis driver TROP2. **A** Western blot of CRISPR/Cas9-mediated ATF2-KO cells and their corresponding parental HCT116 and HT29 cells. ATF2-KO was determined using N- and C-terminal ATF2 antibodies. Representative blots of at least two independent experiments are shown. **B** Heatmap of the HCT116 ATF2-KO gene signature of 26 differentially expressed genes (adjusted *P* < 0.01 and absolute (log2(FC) > 1)) identified by NanoString gene expression analysis (*n* = 3). Heatmap scale: low (blue) to high (red) relative gene expression. **C** Volcano plot for differential expression analysis of HCT116 ATF2-KO versus WT cells. Only genes with an absolute (log2(FC)) < 10 are shown in the plots; red dots: signature of 26 differentially expressed genes (adjusted *P* < 0.01 and absolute (log2(FC) > 1)) with the top five most deregulated genes indicated. **D** RT-qPCR analysis of *TROP2* mRNA levels in parental HCT116 and HT29 cells and their ATF2-KO clones normalized to GAPDH and relative to the corresponding WT controls. Data of three independent experiments are shown as mean ± SEM (***P < 0.001, Mann‐Whitney test). **E** Western blot of TROP2 in HCT116, HT29, and ATF2-KO cells. Representative blots of at least two independent experiments are shown

To identify the gene signature associated with ATF2 loss, we depleted *ATF2* in the two heterogeneous CRC cell lines HCT116 and HT29 (Supplementary Table 3) [16–18] using CRISPR/Cas9, resulting in two ATF2-knockout (KO) clones per cell line (HCT116: F9, E5; HT29: B5, F10) (Fig. 2A, Supplementary Fig. 2A–C). Then, we conducted NanoString gene expression analysis of wildtype (WT) HCT116 and ATF2-KO cells. Of the 740 analyzed transcripts involved in various steps of cancer progression, we focused on genes that were significantly deregulated in both HCT116 ATF2-KO clones compared to the wildtype (adjusted *P* < 0.01 and absolute (log2(FC) > 1)). These criteria resulted in a set of 26 differentially expressed genes (Fig. 2B, C, Supplementary Fig. 2D, and Supplementary Table 4). STRING analysis, a biological database to predict protein–protein interactions, reflected limited experimental knowledge of the identified proteins (Supplementary Fig. 2E). DAVID analysis for functional enrichment based on the 740 panel genes indicated that ATF2 is involved in morphogenesis, migration, and protein phosphorylation pathways (Supplementary Fig. 2F and Supplementary Table 5). Notably, real-time (RT)-qPCR verified the downregulation of the ATF2 target gene *SOX9*, stem cell and EMT-related genes *CD44*, *TWIST1*, protein kinase-encoding *AKT3*, and the DNA-binding inhibitor *ID1* (Supplementary Fig. 2G)*.* Two other EMT-associated genes, *ZEB1* and *E-Cadherin*, were not dysregulated in NanoString analysis (our GSE172488). Interestingly, we observed an upregulation of the ECM-interacting protein *VCAN* and the metastasis promoter *TROP2* (Fig. 2D and Supplementary Fig. 2G). In addition, alterations in *CD44*, *ID1*, and *TROP2*, expression were also validated in the other CRC cell line, HT29 and its corresponding ATF2-KO clones (Fig. 2D and Supplementary Fig. 2G), with *SOX9* being unchanged and *VCAN* and *TWIST1* being undetectable. We selected *TROP2*, also known as *TACSTD2*, for further analysis because (i) its overexpression in CRC has been linked to poor prognosis and high metastatic burden [19], and (ii) our in silico analysis identified several ATF2- and AP1-binding sites in the *TROP2* promoter (Supplementary Table 4). Consistent with elevated *TROP2* transcripts, TROP2 protein levels were increased in both CRC cell lines with ATF2-KO (Fig. 2E), thus supporting a possible novel link between TROP2 and ATF2.

### Intratumoral heterogeneity in primary tumors and liver metastasis

**Fig. 3:**
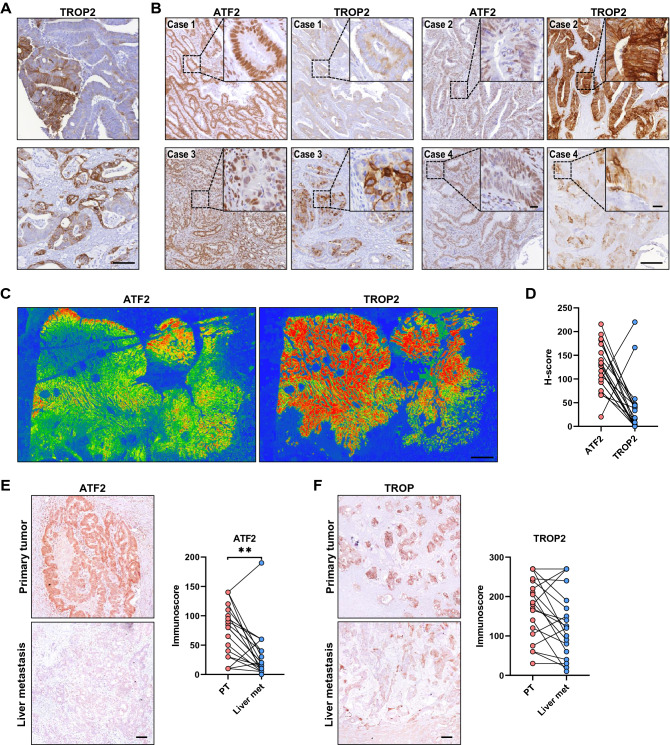
Primary tumors and liver metastasis reveal high intratumoral heterogeneity for ATF2 and TROP2. **A** Representative images of TROP2-stained whole human CRC sections (*n* = 55). Scale: 100 µm. **B** Representative images of ATF2- and TROP2-stained whole human CRC sections (*n* = 55). Overview image, scale: 200 μm; insert, scale: 20 μm. **C** Representative heatmaps for ATF2- and TROP2-stained whole human CRC sections (*n* = 20). Scale: 2 mm. Holes: positions of TMA punches. Color scale: red to green color, high to low staining intensity. **D** H-score of ATF2- and TROP2-stained whole human CRC sections (*n* = 20). ATF2- (**E**) and TROP2-stained (**F**) whole human CRC sections of primary tumors (PT) and liver metastasis (liver met; *n* = 19) and corresponding immunoscore evaluation (**P < 0.01, Wilcoxon test). Scale: 100 µm

Next, we investigated ATF2 and TROP2 expression in whole tissue slices of our CRC TMA cohort. Despite the high ITH of both markers (Figs. 1D and 3A–C, Supplementary Fig. 3A), we could observe an inverse correlation between ATF2 and TROP2 expression in the majority of cases (Fig. 3B; additional cases are given in Supplementary Fig. 3A). When correlating each single ATF2 and TROP2 probe with each other in a public data set GSE41258, we mostly observed a negative correlation, with some of the correlations being statistically significant (Supplementary Table 6). Addressing the ITH in more detail, heatmaps for TROP2-stained CRC sections were generated and compared with the ATF2 heatmaps (Figs. 1D, 3C). The H-score profile for ATF2 and TROP2 of the 20 cases is given in Fig. 3D, and patient-wise intensity profiles in Supplementary Fig. 3B, verifying the ITH and inverse correlation between both markers.

Interestingly, high ITH for ATF2 and TROP2 was also visible in liver metastasis (Fig. 3E, F and Supplementary Fig. 4A, B). Liver metastasis showed significantly lower ATF2 and rather diverse TROP2 expression compared to their primary tumors (Fig. 3E, F, Supplementary Table 7), whereas the expression levels of both markers in the 12 available lymph node metastasis remained nearly unchanged (Supplementary Fig. 4C, Supplementary Table 7). The difference in our observations compared to Guerra et al. [20] who found an increase of TROP2 in metastatic lesions could be explained by the usage of different antibodies, the inclusion of a high number of rectal cancer, and the scoring approach. Otherwise, when analyzing the in silico dataset GSE41258 comparing the gene expression of *TROP2* and *ATF2* in non-paired 182 primary tumors and 47 liver metastasis, both markers showed a probe-dependent high variance in gene expression scores in both cohorts (Supplementary Fig. 4D).

### The JNK-ATF2 axis regulates *TROP2* expression

**Fig. 4:**
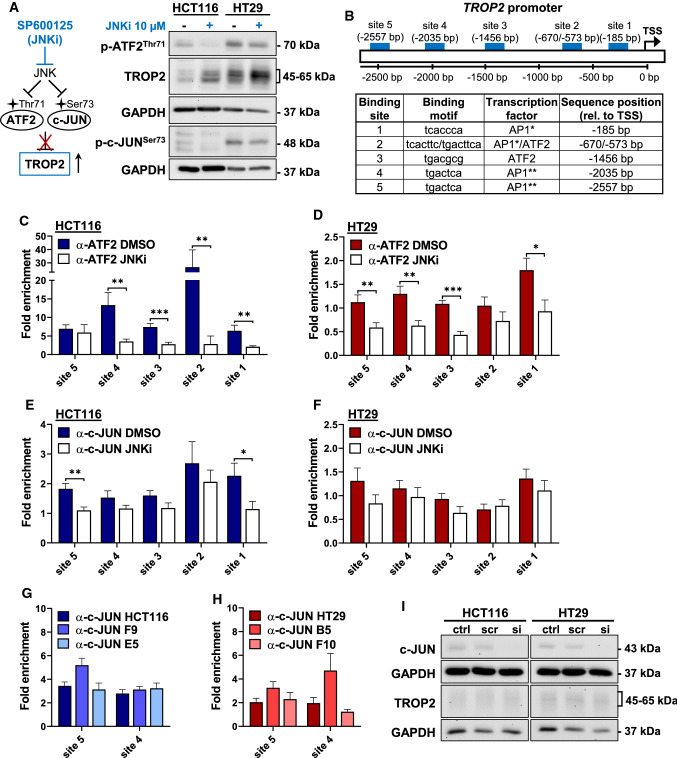
TROP2 levels are regulated by ATF2 via the JNK pathway. **A** *Left*: Schematic illustration of the proposed JNK/ATF2/TROP2 pathway indicating the loss of *TROP2* repression upon JNK inhibition. *Right*: Western blot of HCT116 and HT29 cells treated with either the JNK inhibitor (JNKi) SP600125 (10 µM, in DMSO) or DMSO (−) for 24 h. Representative blots of at least two independent experiments are shown. **B** Illustration of the investigated ATF2- and AP1-binding sites in the *TROP2* promoter. TSS: transcription start site. ChIP-qPCR of HCT116 (**C**, **E**) and HT29 (**D**, **F**) cells treated with either DMSO or the JNK inhibitor (JNKi; 10 µM, in DMSO) SP600125 for 24 h. Fold enrichment of ATF2 (**C**, **D**) and c-JUN (**E**, **F**) was determined over IgG control. Data of independent experiments are given as mean ± SEM (n = 2–3; *P < 0.05, **P < 0.01, ***P < 0.001, Mann–Whitney test). ChIP-qPCR of HCT116 (**G**) and HT29 (**H**) cells and their corresponding ATF2-KO clones at the two consensus AP1-binding sites 4 and 5. Fold enrichment of c-JUN was determined over IgG control. Data of independent experiments are given as mean ± SEM (*n* = 2–3). **I** Western blot of HCT116 and HT29 cells after 48 h of treatment with either *c-JUN*-specific (si) or non-targeting (scr) RNAi. Controls (ctrls) remained untreated. Representative blots of two independent experiments are shown

To determine whether ATF2 regulates *TROP2* expression, we aimed to modulate the upstream mitogen-activated protein kinase JNK, which not only regulates ATF2 but also its AP1 dimerization partner c-JUN [21]. Treatment of HCT116 and HT29 cells with JNK inhibitor SP600125 simultaneously reduced p-ATF2^Thr71^ and p–c-JUN^Ser73^ levels, resulting in increased TROP2 protein levels (Fig. 4A). Similarly, JNK inhibition increased TROP2 expression in all ATF2-KO clones (Supplementary Fig. 5).

Since the *TROP2* promoter harbors several ATF2- and AP1-binding sites (Fig. 4B), we investigated whether ATF2/c-JUN heterodimers directly repressed *TROP2* transcription. Chromatin immunoprecipitation (ChIP) against ATF2 and c-JUN in HCT116 and HT29 cells after JNK inhibition revealed significantly reduced binding of ATF2 to the *TROP2* promoter (Fig. 4C, D), whereas the decrease in c-JUN binding was less pronounced and even partly below the IgG controls (Fig. 4E, F). Notably, ChIP for endogenous c-JUN did not show any differences in c-JUN binding at the two consensus AP1 sites between ATF2-WT and -KO cells (Fig. 4G, H), suggesting that c-JUN-mediated transactivation efficiency cannot explain the remarkable differences in *TROP2* expression. Consistently, transient *c-JUN* silencing in HCT116 and HT29 cells did not affect TROP2 protein expression (Fig. 4I).

### ATF2 loss leads to characteristic cytoskeleton-associated growth pattern in vitro

**Fig. 5:**
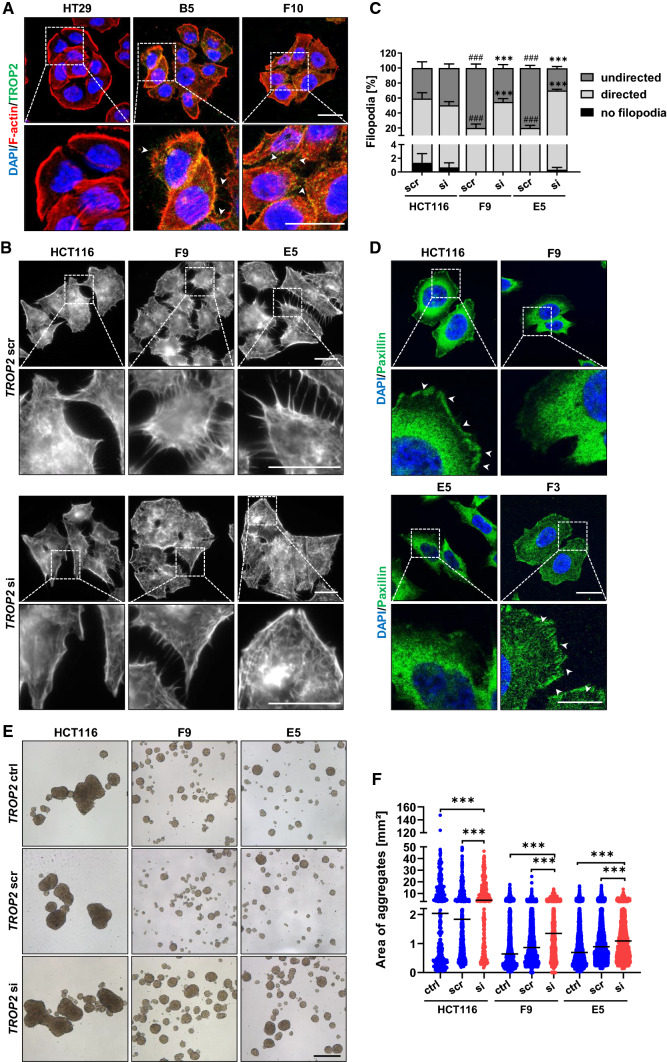
TROP2 is localized in filopodia and mediates cell-to-cell adhesion. **A** Confocal imaging of phalloidin- and TROP2-stained HT29 and ATF2-KO cells showing irregular cell patterning and TROP2 localization in filopodia (arrowheads) upon ATF2 loss. Scale: 20 µm. Representative images of two independent experiments are shown. **B** Phalloidin staining of HCT116 and ATF2-KO cells after 48 h of treatment with either *TROP2*-specific (si) or non-targeting (scr) RNAi. Scale: 20 µm. Representative images of three independent experiments are shown. **C** Quantification of filopodia in the HCT116 and ATF2-KO clones after 48 h of *TROP2* silencing (*n* = 3; *TROP2*si: 75–96 images, *TROP2*scr: 83–95 images, with more than 450 cells per condition and cell line). Percentages of filopodia are presented relative to the number of analyzed cells as mean ± SEM (***P < 0.001: si vs. scr, ^###^*P* < 0.001: ATF2-KO scr vs. ATF2-WT scr; two-way ANOVA). **D** Confocal imaging of Paxillin-stained HCT116 WT, ATF2-KO (F9/E5), and ATF2-/TROP2-KO (F3) cells showing the localization of Paxillin in the adherens junctions in TROP2^low^ (HCT116) and TROP2-KO cells (F3). Overview, scale: 20 µm; insert, scale: 10 µm. **E** Anchorage-independent growth assay after *TROP2* silencing in HCT116 and ATF2-KO cells. Cells were untreated (ctrl) or treated with either *TROP2*si or scr for 48 h under attached conditions and further cultured for 72 h under anchorage-independent conditions. Scale: 500 µm. Representative images of two independent experiments are shown. **F** Quantification of aggregate size of *TROP2*-silenced HCT116 and ATF2-KO cells under anchorage-independent growth (***P < 0.001, Mann–Whitney test). Data from two independent experiments are presented as median

To further assess the functional consequences of elevated TROP2 levels, we investigated the growth pattern of ATF2-KO cells in vitro. HCT116 (Supplementary Fig. 6A) and HT29 cells (Fig. 5A) reflected the typical cobblestone-like morphology of epithelial cells with pronounced cortical F-actin accumulation between adjacent cells and at cellular rims, indicating tight cell–cell adhesion. In contrast, HT29 ATF2-KO clones developed TROP2-enriched filopodia-like protrusions (Fig. 5A, and for HCT116 ATF2-KO clones Supplementary Fig. 6A), suggesting a close association between TROP2 and the cytoskeleton. *TROP2* silencing in both HCT116 ATF2-KO clones reduced filopodia number and length, and re-established an epithelial-like phenotype (Fig. 5B, C and Supplementary Fig. 6B). TROP2 has been previously shown to displace focal adhesion kinase (FAK) [22]. To further evaluate the impact of TROP2 on the spatial distribution of cytoskeleton proteins, we evaluated the expression of Paxillin by immunofluorescence in HCT116 WT cells, in the two HCT116 ATF2-KO clones F9 and E5, and in a CRISPR/Cas9-generated TROP2-KO clone of F9 (F3) (Fig. 4D, Supplementary Fig. 6C, D). Indeed, we detected an accumulation of Paxillin in the adherens junctions of these double ATF2/TROP2-KO cells (Fig. 4D). The cytoskeleton marker E-Cadherin did not show any changes in protein expression (Supplementary Fig. 6E, F).

Next, we performed anchorage-independent growth assays and observed that both HCT116 and HT29-derived ATF2-KO clones formed significantly smaller, but viable cell clusters compared to their parental cell lines as shown in PARP Western blot and Calcein staining, respectively (Fig. 5E, F and Supplementary Fig. 6G-I). *TROP2* silencing under de-adhesive conditions (Supplementary Fig. 6 J) in HCT116 and their ATF2-KO cells led to significantly larger cell aggregates (Fig. 5E, F), suggesting a role for TROP2 in tumor cell adhesion.

### Reduced ATF2 levels promote 2D and 3D tumor cell migration in vitro

First, we evaluated TROP2-overexpressing ATF2-KO clones in a 3D spheroid migration assay and showed their enhanced migratory potential (Supplementary Fig. 7A, B). This effect was further confirmed in a 2D wound healing assay (Supplementary Fig. 7C). To validate a potential TROP2 dependency, we performed a transient *TROP2* silencing in HT29 WT (moderate TROP2 levels) and ATF2-KO clone B5 (high TROP2 levels). *TROP2* silencing did not affect the migration of HT29 WT cells, but showed a significant decrease in clone B5 (Supplementary Fig. 7C).

Since transient *TROP2* silencing was not suitable for a long-term spheroid assay, we generated TROP2-KO cells using CRISPR/Cas9 for HT29 WT and its ATF2-KO clone F10 (Supplementary Fig. 7D, E). We confirmed our findings from the 2D experiment in all previous conditions and could show that again the ATF2/TROP2 double KO was accompanied by a reduced migratory potential (Supplementary Fig. 7F). Finally, these findings support previous reports from literature showing a pro-migratory role for TROP2 [20, 23, 24].

### Reduced ATF2 levels trigger tumor cell invasion in vivo

**Fig. 6:**
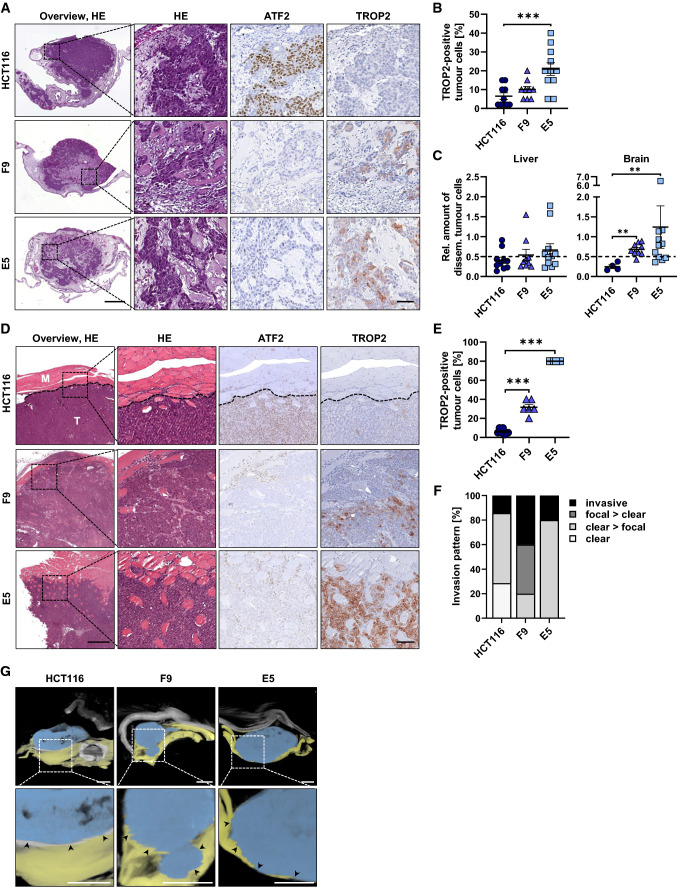
ATF2 loss enhances invasion in different xenograft models. **A** Representative images of HCT116 and ATF2-KO-derived CAM ovografts stained for HE, ATF2, and TROP2 (HCT116: *n* = 10; F9: *n* = 9; E5: *n* = 11). Overview images, scale: 500 µm; enlarged images, scale: 50 µm. (**B**) Quantification of TROP2-positive cells in HCT116 and ATF2-KO-derived ovografts. Data are presented as mean ± SEM (HCT116: *n* = 10; F9: *n* = 9; E5: *n* = 11; ***P < 0.001, Mann‐Whitney test). **C** Relative amount of disseminating tumor cells in the liver (HCT116: *n* = 10; F9: *n* = 9; E5: *n* = 11) and brain (HCT116: *n* = 4; F9: *n* = 9; E5: *n* = 11) of chicken embryos assessed by Alu qPCR on day five post-engraftment. Data are presented as mean ± SEM (**P < 0.01, Mann–Whitney test). Dashed line presents the cut-off for metastasis detection at 0.5. **D** Representative images of HCT116 and ATF2-KO-derived murine subcutaneous xenografts stained for HE, ATF2, and TROP2 (HCT116: *n* = 7; F9: *n* = 6; E5: *n* = 6). Overview images, scale: 500 µm; detailed images, scale: 100 µm;* M* muscle; *T* tumor; dotted line: pushing front margin. **E** Quantification of TROP2-positive cells in murine subcutaneous xenografts derived from HCT116 and ATF2-KO cells. Data are presented as mean ± SEM (HCT116: *n* = 7; F9: *n* = 6; E5: *n* = 6; ***P < 0.001, Mann‐Whitney test). **F** Invasion pattern of murine subcutaneous xenografts derived from HCT116 and ATF2-KO cells. Invasion was classified as “clear” (distinct border between muscle and tumor), “clear > focal” (more clear borders than areas with focal invasions), “focal > clear” (more focal invasions than clear borders), or “invasive” (no clear borders). Percentages of each category are given. Only samples with sufficient surrounding muscle tissue were evaluated (HCT116: *n* = 7; F9: *n* = 5; E5: *n* = 5). **G** Micro-CT analysis of HCT116 and ATF2-KO-derived subcutaneous xenografts. Segmentation of 3D datasets discriminating between tumor (blue) and muscle (yellow) was performed on one representative mouse per group (*n* = 3). Arrowheads indicate muscle invasion. Scale: 2.5 mm

To evaluate a potential TROP2 dependency on hallmarks of tumor aggressiveness, we performed the chicken chorioallantoic membrane (CAM) assay as an in vivo xenograft model pursuing the ethical responsibility to replace, reduce, and refine (3R) animal experiments. Our tumor cell line sets were grafted onto the CAM and their in vivo growth patterns were compared based on hematoxylin/eosin (HE) and IHC staining (Fig. 6A and Supplementary Fig. 8A). We detected the typical microsatellite-unstable tumor pattern in the ovografts of HCT116 cells, with a dense tumor mass and a clearly defined pushing front at the invasive border (Fig. 6A). In contrast, HCT116 ATF2-KO clones displayed more loosely arranged tumor masses lacking a clear pushing front (Fig. 6A). A shift in the growth pattern upon ATF2 loss was also observed in HT29-KO cells (Supplementary Fig. 8A, B), suggesting that tumor cell de-adhesion is increased when ATF2 is lost. CAM experiments with a lower number of HCT116 cells demonstrated that the differences in the growth pattern of HCT116 ATF2-KO clones were not due to biologically relevant differences in proliferation (Supplementary Fig. 8C) as also shown by staining with the proliferation marker Ki67 (Supplementary Fig. 8D, E). All cell lines developed highly proliferative tumors in vivo (median > 70%).

All ATF2-KO ovografts revealed upregulation of TROP2 expression (Fig. 6A, B and Supplementary Fig. 8A, B), further supporting a suppression of TROP2 when ATF2 is expressed. In addition, ATF2^KO^/TROP2^high^ tumor cells showed increased invasion into chicken embryonic organs, as determined by human-specific Alu-PCR (Fig. 6C). The presence of disseminating ATF2-KO tumor cells in the brain of chicken might underline the potential of ATF2-KO cells to spread to multiple and more unusual sites.

The invasive behavior of ATF2-KO cells was further investigated in subcutaneous mouse xenografts using luciferase-labeled HCT116 and ATF2-KO cells (Fig. 6D, E, and Supplementary Fig. 8F, G). These xenografts do not constitute a metastasis model; rather, they allowed us to examine the invasive growth pattern, a prerequisite for metastasis. Indeed, tumors of HCT116 ATF2-KO cells were highly TROP2-positive (Fig. 6D, E) and presented primarily deeper invasion toward the muscle layer (Fig. 6D-F) as supported by micro-CT analysis (Fig. 6G). In contrast, the majority of HCT116-derived tumors had a predominantly cohesive pushing front that clearly segregated tumor cells from the surrounding muscle layer with only minor focal invasions (Fig. 6D and F), suggesting that the loss of ATF2 remarkably alters the invasion pattern.

### TROP2 is a suitable prognostic marker in CRC

**Fig. 7:**
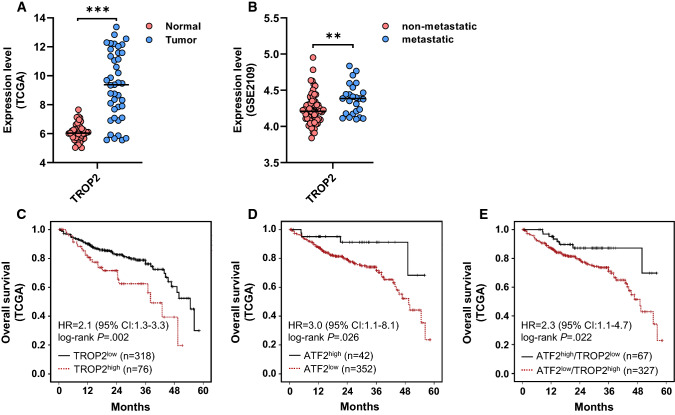
Upregulation of *TROP2* expression in CRC is associated with enhanced tumor aggressiveness and predicts poor patient survival. **A** *TROP2* in normal (*n* = 41) and tumorous colon tissue (*n* = 41; ** *P* < 0.01, *** *P* < 0.001, Welch’s *t* test). The line shows the median. Gene expression data were extracted from the TCGA RNA-seq database (https://www.cancer.gov/tcga). **B** *TROP2* expression in metastatic (*n* = 25) versus non-metastatic primary (*n* = 65) CRC as extracted from the GSE2109 dataset. The line shows the median (**P < 0.01, Welch’s *t* test). Kaplan–Meier plots for overall survival in the TCGA CRC cohort grouped according to their optimal *TROP2* (**C**), *ATF2* (**D**) and combined *TROP2*/*ATF2* (**E**) expression (*n* = 394, log-rank test). *HR* hazard ratio, *CI* confidence interval

**Fig. 8:**
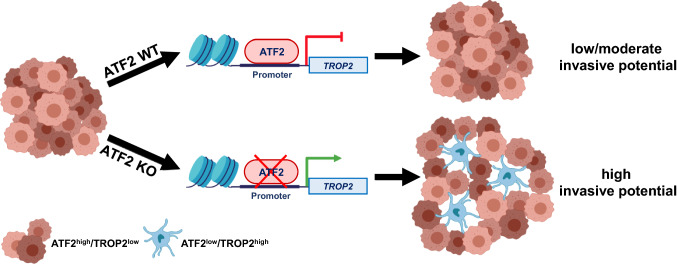
Working model illustrating the ATF2-dependent transcriptional regulation of *TROP2* and its impact on tumor invasiveness. Created with BioRender.com

Based on our data, TROP2 can be considered as a marker of tumor aggressiveness in CRC cell lines. To evaluate the clinical relevance of our findings, we performed several in silico analyses based on gene expression data. First, *TROP2* expression was significantly upregulated in tumors compared to matched normal colon tissue (Fig. 7A). Next, we found that primary tumors with metastasis showed an upregulation of *TROP2* expression in comparison to primary tumors without metastasis (Fig. 7B). When performing survival analysis for TROP2 in the TCGA CRC cohort applying the commonly used median expression score, we did not find any significant prognostic relevance in Kaplan–Meier plots. However, using an optimal score for dichotomizing patients with TROP2^high^ and TROP2^low^ tumors, that considers the best patient separation by survival using the *survminer* algorithm, high *TROP2* levels were associated with shortened overall survival (hazard ratio [HR] 2.1) (Fig. 7C). ATF2 in TCGA without optimal score using median separation showed that ATF2^low^ expressing tumors had a worse prognosis than ATF2^high^ expressing ones (HR 1.6). In the next step, we adapted the Kaplan–Meier analysis for ATF2 using the optimal score approach and this even reinforced the hazard ratio to 3.0. In the case of ATF2, the optimal separation defined a threshold of 11% for patients who had the highest *ATF2* expression (Fig. 7D). This optimal score was not suitable for IHC analysis in our TMA cohort since remarkably more than 11% of patients showed ATF2^high^ expressing tumors. Next, we tested a combined TROP2/ATF2 optimal score. This approach revealed an increased hazard ratio (HR = 2.3) compared to TROP2 alone (HR = 2.1) and was associated with a remarkably reduced overall survival in patients with ATF2^low^/TROP2^high^ status (Fig. 7E). Translationally, these findings suggest that a patient diagnosed with a TROP2^high^ tumor with concomitant ATF2^low^ expression is under high risk for invasion/metastasis (Fig. 8).

## Discussion

In this study, we have systematically investigated the role of ATF2 in CRC invasion. We suggest that the presence of an ATF2-negative tumor cell population is associated with a higher de-adhesion, migration, and invasion potential of tumors. The cancer driver TROP2 has been identified as a novel transcriptional repressive target of ATF2. Although ATF2 loss constitutes a disease-associated condition, ATF2 per se is a rather unsuitable therapeutic target in CRC. Instead, we uncovered TROP2 as a potential novel therapeutic target to inhibit the first step in the metastatic cascade in CRC.

We observed a high intratumoral heterogeneity (ITH) for ATF2 protein expression by immunohistochemistry in our CRC tissue cohort. Possibly, such ITH might be a reason why genes deemed as “non-interesting” have not been deeply investigated in the context of CRC aggressiveness. Such ITH might mask and decisively impact not only the experimental outcomes but similarly also the metastatic spread and consequently patient prognosis. Our findings that ITH might be preserved in liver metastases of colon tumors let us suggest that the existence and the degree of ITH is not random, rather this is a well-orchestrated cellular mechanism to develop the full aggressiveness of a tumor. The monoclonal expansion approach of CRISPR/Cas9-mediated ATF2-KO cells allowed us to abrogate ITH and capture, at least partly, the genetic diversity in the tumor, leading to the identification of a novel regulatory axis between ATF2 and TROP2.

We found that ATF2/AP1 repressed the expression of TROP2 by directly binding to CRE and TRE motifs in the *TROP2* promoter. ChIP experiments revealed that ATF2 homo-/heterodimers were decisive for *TROP2* transcription with a negligible role of c-JUN in *TROP2* promoter binding. This molecular mechanism is a rare example that corroborates the role of the ATF2/AP1 complex in target gene repression. However, given that c-JUN can form AP1 dimers with other bZIP family members such as FOS, and that different AP1 dimers can bind to DNA with different affinities and transactivation efficiencies [25], we cannot fully exclude such interactions at the *TROP2* promoter.

TROP2 is known to be an important cancer driver and therapeutic target [26]. It functions as a transmembrane glycoprotein and is overexpressed in numerous solid cancers [27]. TROP2 was assessed as an independent prognostic marker correlating with poor patient prognosis in CRC [26, 28] and was linked to tumor budding, a marker of increased tumor aggressiveness [29]. Accordingly, a pro-migratory role has already been ascribed to TROP2 in various solid tumor types [30–32]. Our study reveals a novel and important mechanism for the regulation of TROP2 expression via ATF2, mechanistically explaining the increased invasive potential of ATF2-deficient tumor cells.

Additionally, we have revealed a potentially more decisive function of TROP2 in de-adhesion of cancer cells as the starting point of metastasis. TROP2 was localized in long cell protrusions interspersing ATF2-negative cell aggregates, linking its function to the cytoskeleton machinery as recently described [33]. Such filopodia act as sensors for signals, such as chemo-attractants or nutrients. Interestingly, metastatic cells are rich in filopodia-like structures [34]. Recently, TROP2-interacting proteins were linked to matrix degradation, cell shape, motility, and invasion in CRC cells [35]. We found that under adhesion blockade, ATF2-KO cells built only vital single cells or small aggregates, and that transient *TROP2* silencing attenuated this de-adhesive effect, which was accompanied by a loss in cell–cell protrusions. Focusing on focal adhesion kinase (FAK), Trerotola et al. showed that prostate cancer cells silenced for *TROP2* accumulated FAK at focal adhesion sites together with α5β1 integrin [22]. Thus, we studied Paxillin, which is important for the formation of functional adherens junctions, in CRISPR/Cas9-generated TROP2-KO cells of HCT116 ATF2-KO clone F9. Indeed, we observed a clear accumulation of Paxillin in the adherens junctions when TROP2 was lost. Since epithelial proteins E-Cadherin or EpCAM were not altered in their levels, we suggest that it is rather the spatial dysregulation of the TROP2 complex members at the cell membrane than a TROP2-mediated alteration in protein amounts of the complex partners. Correspondingly, in vivo, TROP2-overexpressing xenografts grew as loosely packed tumors and a disturbed pattern of cellular contacts was further reflected by the deregulation of the adhesion molecules *MCAM* and *ICAM* in a NanoString analysis. Interestingly, the higher migration and invasiveness in TROP2-overexpressing ATF2-KO cell lines were not associated with robust EMT signs as already shown in three different tumor entities by Remsik et al. for breast and prostate cancer [36], and Guerra et al. for colorectal cancer [20]. In the NanoString analysis, EMT markers, such as *CD44* and *TWIST1*, were even down-regulated, and *ZEB1*, *SNAI2*, and *E-Cadherin* levels were unchanged, supporting the findings of Guerra et al. who described an EMT-less invasion in their TROP2-overexpressing metastatic cells [20]. Thus, we suggest that ATF2 loss seems to reinforce the epithelial differentiation.

Using our own and public clinical datasets incorporating both RNA sequencing and IHC data, we have shown that low ATF2 expression could significantly predict high-risk CRC patients. Moreover, TROP2^high^ human tumors that were concomitantly ATF2^low^ could further increase the hazard ratio suggesting that a combination of ATF2^low^/TROP2^high^ could serve as a suitable biomarker for susceptibility to highly invasive tumors. The use of the survminer algorithm to optimally separate between the prognostic groups might be more reliable than the separation by the median score allowing a more robust comparison between different studies.

Our observations have high clinical relevance. Unraveling the basic mechanisms of the first steps in the metastatic process, i.e., the de-adhesion and invasion of cancer cells, can open up novel therapeutic approaches for successful interventions in CRC. Considering several ongoing clinical trials [37] and the recently FDA-approved drug sacituzumab govitecan-hziy, which combines a TROP2-directed antibody and a topoisomerase inhibitor [38, 39], TROP2 holds promise as a marker for tumor aggressiveness in CRC patients.

### Supplementary Information

Below is the link to the electronic supplementary material.

Supplementary file1 (DOCX 142 KB)

Supplementary file2 (PDF 594 KB)

Supplementary file3 (PDF 424 KB)

Supplementary file4 (PDF 497 KB)

Supplementary file5 (PDF 1576 KB)

Supplementary file6 (PDF 22 KB)

Supplementary file7 (PDF 218 KB)

Supplementary file8 (PDF 102 KB)

Supplementary file9 (PDF 656 KB)

## Data Availability

All data relevant to the study are included in the article or uploaded as supplementary information. NanoString gene expression data supporting the conclusions of this article are deposited in NCBI's Gene Expression Omnibus (GEO), accession number GSE172488. Sequencing data are available upon reasonable request directed to Regine Schneider-Stock (regine.schneider-stock@uk-erlangen.de).
