# Supplementary material for: ATF2 loss promotes tumor invasion in colorectal cancer cells via upregulation of cancer driver TROP2
Source: Cell Mol Life Sci. 2022 Jul 15;79(8):423. doi: 10.1007/s00018-022-04445-5 (PMC9287261; doi:10.1007/s00018-022-04445-5)
Supplement: Supplementary file 1 — Supplementary file1 (DOCX 142 KB) [file 18_2022_4445_MOESM1_ESM.docx]

**Supplemental Materials and Methods**

**Huebner et al.**

## ATF2 loss promotes tumor invasion in colorectal cancer cells via upregulation of cancer driver TROP2

Kerstin Huebner^1^, Katharina Erlenbach-Wuensch^2^, Jan Prochazka³, Ilir Sheraj^4^, Chuanpit Hampel^1^, Blanka Mrazkova^3^, Tereza Michalcikova^3^, Jolana Tureckova³; Veronika Iatsiuk³, Anne Weissmann^1^, Fulvia Ferrazzi^2,5^, Philipp Kunze^1^, Enise Nalli^1^, Elisabeth Sammer^1^, Annemarie Gehring^1^, Marie M. Cheema^3^, Markus Eckstein^2^, Eva-Maria Paap^1^, Agnes Soederberg^1^, Corinna Fischer^1^, Sushmita Paul^7^, Vijayalakshmi Mahadevan^8^, Benardina Ndreshkjana^1^, Melanie A. Meier^9^, Susanne Muehlich^9^, Carol I. Geppert^2^, Susanne Merkel^10^, Robert Grutzmann^10,11^, Adriana Roehe^12^, Sreeparna Banerjee^4^, Arndt Hartmann^2,11^, Radislav Sedlacek^3^, Regine Schneider-Stock^1,11*^

^*^Correspondence: Regine Schneider-Stock, PhD, Experimental Tumorpathology, FAU Erlangen-Nürnberg, Universitätsstraße 22, 91054 Erlangen, Germany; regine.schneider-stock@uk-erlangen.de

**Supplementary Figure legends**

**Supplementary Tables**

**Supplementary Material and Methods**

# Supplementary Figure legends

## Supplementary Fig. 1 ATF2 expression reveals enhanced intratumoral heterogeneity

(**A**) ATF2 score based on ATF2 IHC staining on a tissue microarray (TMA) of CRC patients. Representative images of ATF2 IHC-stained punch biopsy samples indicate low (1), moderate (2) and strong (3) intensity for ATF2 expression. Scale: 100 µm.

(**B**) Additional representative heatmaps and corresponding ATF2 IHC staining of CRC samples (n=20). Heatmap scale: 2 mm, IHC overview, scale: 200 µm; insert, scale: 20 µm; holes: positions of TMA punches; red arrowheads: ATF2-negative cells.

## Supplementary Fig. 2 CRISPR/Cas9-mediated ATF2 knockout in HCT116 and HT29 colorectal cancer cells.

(**A**) For CRISPR/Cas9-mediated gene editing, single guide RNA (sgRNA) targeting exon 5 of human *ATF2* (NM_001880.4) was designed and introduced into pX330-U6-Chimeric_BB-CBh-hSpCas9 at the *Bbs*I site.

(**B**) Schematic illustration of the *ATF2* gene (upper panel) and the targeted region (exon 5, lower panel) for CRISPR gene editing. PAM: protospacer adjacent motif.

(**C**) Western blot of h*Sp*Cas9 in parental HCT116 and HT29 cells 24 h after transfection. Asterisks indicate cropped images at this position with corresponding samples being loaded and analyzed on the same SDS-PAGE gel and Western blot.

(**D**) Heatmap of sample-to-sample distances on all genes of the NanoString gene expression array. Color scale: the darker the blue, the closer two samples are.

(**E**) STRING analysis (version 11.0) of the 26 significantly deregulated genes of the ATF2-KO signature. ATF2 was additionally added to the set as marked in red and by an asterisk.

(**F**) Analysis of the HCT116 ATF2-KO signature using DAVID (Database for Annotation, Visualization and Integrated Discovery) gene-annotation enrichment analysis of Gene Ontology (GO) (*P*<0.05, Fisher’s exact test).

(**G**) RT-qPCR evaluation of significantly altered genes identified in NanoString gene expression analysis in HCT116 (blue panel) and HT29 (red panel) cells normalized to GAPDH and relative to the corresponding WT controls. Data of three independent experiments are shown as mean ± SEM (**P*<0.05; ****P*<0.001, Mann-Whitney test).

## Supplementary Fig. 3 Intratumoral heterogeneity for ATF2 and TROP2 in primary tumors of CRC.

(**A**) Additional representative images of ATF2- and TROP2-stained whole human CRC sections (n=55). Overview image, scale: 200 µm; insert, scale: 20 µm.

(**B**) Patient-wise intensity score profiles (AI-based) for ATF2 and TROP2 (n=20), representing the distribution of tumor cells with intensities from 0-3 and given as percentage of total scored cells.

## Supplementary Fig. 4 Intratumoral heterogeneity for ATF2 and TROP2 in liver and lymph node metastasis of CRC.

(**A**, **B**) Additional representative images of ATF2- (**A**) and TROP2-stained (**B**) liver metastasis sections (n=19). Scale: 100 µm.

(**C**) ATF2- and TROP2-stained whole human sections of CRC lymph node metastasis (*left*) and the corresponding paired immunoscores (*right*; n=12; **P*<0.05, ***P*<0.01, Wilcoxon test). The line shows the median. PT: primary tumor, LN met: lymph node metastasis, Liver met: liver metastasis. Scale: 200 µm.

(**D**) TROP2 and ATF2 expression in non-paired primary tumors and liver metastasis as extracted from the GSE41258 dataset. The line shows the median (**P*<0.05, ****P*<0.001, Mann-Whitney test).

## Supplementary Fig. 5 ATF2 inversely regulates TROP2.

Western blot of TROP2 in JNK-inhibited HCT116, HT29 and ATF2-KO cells. Cells were treated with either JNK inhibitor (JNKi) SP600125 (10 µM, in DMSO) or DMSO (-) for 24 h. Representative blots of two additional independent experiments are shown.

## Supplementary Fig. 6 ATF2 loss results in de-adhesion phenotype.

(**A**) Filopodia formation (arrowheads) upon ATF2 loss as shown by confocal microscopy of phalloidin-stained HCT116 and ATF2-KO cells. Scale: 20 µm. Representative images of at least two independent experiments are presented.

(**B**) *Left*: Flow chart of the evaluation criteria applied for the quantification of filopodia. Only fully-visible and properly stained cells were categorized into one of the following groups: “no filopodia” (cells that do not show any filopodia at the outer cell membrane), “directed filopodia” (cell with filopodia that are between or directed towards adjacent cells), and “undirected filopodia” (cells with filopodia that do not show any directionality independent of any other directed filopodia). *Right*: Representative images for the quantification of filopodia.
Scale: 25 µm.

(**C**) Western blot for TROP2 and its homolog EpCAM in ATF2-KO cells F9 and its corresponding ATF2-/TROP2-KO clone F3. Representative blots of two independent experiments are shown.

(**D**) Western blot for h*Sp*Cas9 in parental ATF2-KO cells F9 24 h after transfection. Representative blots of two independent experiments are shown.

(**E**, **F**) Western blot for E-Cadherin in HCT116 (**E**) and HT29 WT cells (**F**) and their corresponding ATF2-KO clones. Representative blots of two independent experiments are shown.

(**G**) Quantification of aggregate size of HCT116, HT29, and ATF2-KO cells after 96 h of anchorage independent growth (****P*<0.001, Mann-Whitney test). Data are presented as mean ± SEM (n=2).

(**H**) Western blot for PARP of HCT116 and ATF2-KO clones collected at 96 h after anchorage independent growth. Representative blots of two independent experiments are shown.

(**I**) Anchorage independent growth assay in HT29 and ATF2-KO cells and corresponding assessment of cell viability via calcein staining (2 µM) at 96 h post-seeding. Scale: 200 µm. Representative images of two independent experiments are shown.

**(J**) RT-qPCR analysis of *TROP2* mRNA levels in HCT116 and ATF2-KO cells after 48 h of treatment with either *TROP2*-specific (si) or non-targeting (scr) RNAi and additional 72 h of anchorage independent growth. Controls (ctrls) remained untreated. Data are presented as mean ± SEM relative to corresponding scr conditions (***P*<0.01, Mann-Whitney test; n=2).

## Supplementary Fig. 7 ATF2 loss promotes tumor cell migration in 2D and in 3D.

(**A**) Quantification of relative migration area of HCT116 WT and ATF2-KO 3D spheroids, normalized to 0 h (***P*<0.01; ****P*<0.001, two-way ANOVA). Data are presented as mean ± SEM (n≥2).

(**B**) Quantification of relative migration area of HT29 WT and ATF2-KO 3D spheroids, normalized to 0 h (****P*<0.001, two-way ANOVA). Data are presented as mean ± SEM (n=2).

(**C**) Wound healing assay of HT29 and its ATF2-KO cells (B5) after 48 h of *TROP2* silencing (***P*<0.01; ****P*<0.001, two-way ANOVA). Proliferation was inhibited by 1.0 µg/ml mitomycin C treatment. The remaining gap area was normalized to gap area at 0 h. Data are presented as mean ± SEM (n=2).

(**D**) Western blot for TROP2 and its homolog EpCAM in HT29 WT cells and its corresponding TROP2-KO clone A4, as well as in ATF2-KO cells F10 and its corresponding ATF2-/TROP2-KO clone G3. Representative blots of two independent experiments are shown.

(**E**) Western blot for h*Sp*Cas9 in parental HT29 WT and ATF2-KO cells F10 24 h after transfection. Representative blots of two independent experiments are shown.

(**F**) Quantification of relative migration area of 3D spheroids from HT29 WT cells and its corresponding TROP2-KO clone A4, and from ATF2-KO F10 and its corresponding
ATF2-/TROP2-KO clone G3, normalized to 0 h (****P*<0.001, Mann‐Whitney test). Data are presented as mean ± SEM (n≥2).

## Supplementary Fig. 8 Loss of ATF2 alters the *in vivo* growth pattern in different xenograft models.

(**A**) Representative images of HT29 and ATF2-KO-derived CAM ovografts (HT29: n=7;
B5: n=14; F10: n=12) stained for HE, ATF2, and TROP2. Overview images, scale: 500 µm; enlarged images, scale: 50 µm.

(**B**) Quantification of TROP2-positive cells in HT29 and ATF2-KO-derived ovografts. Data are presented as mean ± SEM (****P*<0.001, Mann‐Whitney test; HT29: n=7; B5: n=14; F10: n=12).

(**C**) HE images of HCT116 CAM tumors. Tumors were inoculated with a reduced cell number of 0.5x10^6^ cells (n=5). Scale: 500 µm.

(**D**, **E**) Representative images of CAM ovografts stained for Ki67 (**D**, HCT116; **E**, HT29; scale:

100 μm) and quantification of Ki67-positive tumor cells. Data are presented as median (***P<0.001, Mann-Whitney test; HCT116: n = 10; F9: n = 9; E5: n = 11, HT29: n = 7; B5:
n = 14; F10: n = 12).

(**F, G)** Bioluminescence imaging (BLI) of subcutaneous tumors derived from luciferase-labeled HCT116 and ATF2-KO cells. Representative mice of each group were chosen to demonstrate time course of BLI (in photons/s/mm/sq; **F**). Asterisks indicate animals sacrificed for welfare reasons on particular day/stage of tumor progression. Quantification of bioluminescence signals over time (in photons/s; **G**). Data are presented as mean  ±  SEM (n_initial_=10; **P*<0.05, ***P*<0.01: F9 vs. HCT116, ^#^*P*<0.05: E5 vs. HCT116; two-way ANOVA).

# Supplementary Tables

**Supplementary Table 1. Patient characteristics (n=332) and association with immunohistochemical ATF2 expression.**

| **Feature** | **n (%)** | **ATF2 expression <median** | **ATF2 expression >median** | ***P*-value** |
| --- | --- | --- | --- | --- |
| Age median (range) (years) | 67 (22-91) | 68 (39-89) | 66.5 (22-91) | .822 |
| Sex |  |  |  |  |
| Male | 195 (58.7) | 102 (52.3) | 93 (47.7) |  |
| Female | 137 (41.3) | 64 (46.7) | 73 (53.3) | .316 |
| Histological type |  |  |  |  |
| Adenocarcinoma | 298 (89.8) | 150 (50.3) | 148 (49.7) |  |
| Mucinous adenocarcinoma | 34 (10.2) | 16 (47.1) | 18 (52.9) | .717 |
| pT |  |  |  |  |
| pT1 | 17 (5.1) | 5 (29.4) | 12 (70.6) |  |
| pT2 | 58 (17.5) | 31 (53.4) | 27 (46.6) |  |
| pT3 | 190 (57.2) | 91 (47.9) | 99 (52.1) |  |
| pT4 | 67 (20.2) | 39 (58.2) | 28 (41.8) | .151 |
| pN |  |  |  |  |
| pN0 | 195 (58.7) | 88 (45.1) | 107 (54.9) |  |
| pN1 | 82 (24.7) | 45 (54.9) | 37 (45.1) |  |
| pN2 | 55 (16.6) | 33 (60.0) | 22 (40.0) | .089 |
| cM/pM |  |  |  |  |
| M0 | 260 (78.3) | 121 (46.5) | 139 (53.5) |  |
| M1a (solitary organ) | 41 (12.3) | 22 (53.7) | 19 (46.3) |  |
| M1b (without peritoneum) | 5 (1.5) | 5 (100.0) | 0 (0.0) |  |
| M1c (peritoneum +/- others) | 26 (7.8) | 18 (69.2) | 8 (30.8) | **.013*** |
| Stage (UICC) |  |  |  |  |
| Stage I | 62 (18.7) | 27 (43.5) | 35 (56.5) |  |
| Stage II | 122 (36.7) | 55 (45.1) | 67 (54.9) |  |
| Stage III | 76 (22.9) | 39 (51.3) | 37 (48.7) |  |
| Stage IV | 72 (21.7) | 45 (62.5) | 27 (37.5) | .080 |
| Tumor site^#^ |  |  |  |  |
| Right colon | 153 (46.1) | 82 (53.6) | 71 (46.4) |  |
| Left colon | 179 (53.9) | 84 (46.9) | 95 (53.1) | .226 |
| Grading |  |  |  |  |
| Low grade (G1,2) | 220 (66.3) | 103 (46.8) | 117 (53.2) |  |
| High grade (G3,4) | 112 (33.7) | 63 (56.3) | 49 (43.8) | .104 |
| Lymphatic invasion |  |  |  |  |
| L0 | 219 (66.0) | 102 (46.6) | 117 (53.4) |  |
| L1 | 113 (34.0) | 64 (56.6) | 49 (43.4) | .082 |
| Venous invasion |  |  |  |  |
| V0 | 307 (92.5) | 151 (49.2) | 156 (50.8) |  |
| V1 | 25 (7.5) | 15 (60.0) | 10 (40.0) | .298 |

^#^ right sided: caecum to the proximal two thirds of the transverse colon; left sided: distal third of the transverse colon to the sigmoid colon; **P*<0.05; statistical analysis was performed using the χ² test.

**Supplementary Table 2. Prognostic significance (cancer-related survival) of clinicopathological parameters as determined by Uni- and Multivariate Cox regression analysis.**

|  |  | **Univariate analysis** | | | **Multivariate analysis** | | |
| --- | --- | --- | --- | --- | --- | --- | --- |
| **Feature** | **n** | **HR** | **95% CI** | ***P*-value** | **HR** | **95% CI** | ***P*-value** |
| ATF2 |  |  |  |  |  |  |  |
| ATF2 expression<median | 166 | 1.0 |  |  | 1.0 |  |  |
| ATF2 expression>median | 166 | 0.6 | 0.5-0.9 | **.003**** | 0.8 | 0.6-1.1 | .102 |
| Sex |  |  |  |  |  |  |  |
| Male | 193 | 1.0 |  |  |  |  |  |
| Female | 139 | 0.8 | 0.6-1.1 | .145 |  |  |  |
| Histological type |  |  |  |  |  |  |  |
| Adenocarcinoma | 298 | 1.0 |  |  |  |  |  |
| Mucinous adenocarcinoma | 34 | 1.1 | 0.7-1.9 | .536 |  |  |  |
| pT |  |  |  |  |  |  |  |
| pT1 | 17 | 1.0 |  |  | 1.0 |  |  |
| pT2 | 58 | 1.9 | 0.7-4.9 | .180 | 1.7 | 0.6-4.4 | .273 |
| pT3 | 190 | 2.3 | 0.9-5.6 | .073 | 1.4 | 0.6-3.5 | .461 |
| pT4 | 67 | 4.7 | 1.9-11.9 | **.001**** | 2.1 | 0.8-5.7 | .116 |
| pN |  |  |  |  |  |  |  |
| pN0 | 195 | 1.0 |  |  | 1.0 |  |  |
| pN1 | 82 | 1.5 | 1.1-2.2 | **.018*** | 1.0 | 0.7-1.5 | .953 |
| pN2 | 55 | 4.0 | 2.8-5.8 | **<.001***** | 1.5 | 0.9-2.4 | .095 |
| cM/pM |  |  |  |  |  |  |  |
| M0 | 260 | 1.0 |  |  | 1.0 |  |  |
| M1a (solitary organ) | 41 | 4.7 | 3.1-7.0 | **<.001***** | 3.9 | 2.5-6.1 | **<.001***** |
| M1b (without peritoneum) | 5 | 6.5 | 2.6-16.0 | **<.001***** | 3.2 | 1.2-8.6 | **.018*** |
| M1c (peritoneum +/- others) | 26 | 7.9 | 5.1-12.4 | **<.001***** | 4.1 | 2.4-7.1 | **<.001***** |
| Stage (UICC) |  |  |  |  |  |  |  |
| Stage I | 62 | 1.0 |  |  |  |  |  |
| Stage II | 122 | 1.0 | 0.6-1.6 | .986 |  |  |  |
| Stage III | 76 | 1.2 | 0.7-2.0 | .440 |  |  |  |
| Stage IV | 72 | 6.1 | 3.8-9.6 | **<.001***** |  |  |  |
| Tumor site^#^ |  |  |  |  |  |  |  |
| Right colon | 153 | 1.0 |  |  |  |  |  |
| Left colon | 179 | 0.8 | 0.6-1.1 | .275 |  |  |  |
| Grading |  |  |  |  |  |  |  |
| Low grade (G1,2) | 220 | 1.0 |  |  | 1.0 |  |  |
| High grade (G3,4) | 112 | 1.6 | 1.2-2.1 | **.002**** | 1.1 | 0.8-1.6 | .539 |
| Lymphatic invasion |  |  |  |  |  |  |  |
| L0 | 219 | 1.0 |  |  | 1.0 |  |  |
| L1 | 113 | 2.5 | 1.9-3.3 | **<.001***** | 1.5 | 1.0-2.2 | **.044*** |
| Venous invasion |  |  |  |  |  |  |  |
| V0 | 307 | 1.0 |  |  | 1.0 |  |  |
| V1 | 25 | 2.0 | 1.2-3.3 | **.005**** | 1.1 | 0.6-1.8 | .814 |

^#^ right sided: caecum to the proximal two thirds of the transverse colon; left sided: distal third of the transverse colon to the sigmoid colon; HR: hazard ratio; * *P*<0.05; ** *P*<0.01; *** *P*<0.001.

**Supplementary Table 3. Mutation status of colorectal cancer cell lines HCT116 and HT29.**

| **Cell line** | **MSI status** | **KRAS** | **BRAF** | **PIK3CA** | **PTEN** | **TP53** | **APC** | **CTNNB1** |
| --- | --- | --- | --- | --- | --- | --- | --- | --- |
| HCT116 | MSI | G13D | WT | H1047R | WT | WT | WT | S45del |
| HT29 | MSS | WT | V600E | P449T | WT | R273H | E853*;  T1556fs*3 | WT |

MSI, microsatellite instability; MSS, microsatellite stable; KRAS, KRAS Proto-Oncogene; BRAF, B-Raf Proto-Oncogene; PIK3CA, Phosphatidylinositol-4,5-Bisphosphate 3-Kinase Catalytic Subunit Alpha; PTEN, Phosphatase And Tensin Homolog; TP53, Tumor Protein 53; APC, Adenomatous Polyposis Coli Protein; CTNNB1, Catenin Beta-1; fs, frameshift; WT, wildtype; * nonsense mutation.

## Supplementary Table 4. 26 deregulated genes in HCT116 ATF2-KO cells compared to HCT116 cells.

| **Gene** | **RefSeq No.** | **log2(FC)** | | | **p-value** | | **adjusted p-value** | | **ATF2 (+)/AP-1^a^ (++) motif** | | **Consulted databases and datasets***^b^* |
| --- | --- | --- | --- | --- | --- | --- | --- | --- | --- | --- | --- |
| *A: Upregulated genes* | | |  | | |  | | | | | |
| *P3H2* | NM_018192.2 | 4.16 | | | 5.56E-05 | | 9.57E-04 | | +/++ | | TRANSFAC (TFTs)/ChEA/ENCODE (TFTs)/JASPAR |
| *PDGFC* | NM_016205.2 | 1.47 | | | <1E-16 | | <1E-16 | | ++ | | JASPAR |
| *SEMA3E* | NM_012431.1 | 1.15 | | | 3.41E-05 | | 6.15E-04 | | +/++ | | JASPAR |
| *SYK* | NM_003177.3 | 1.76 | | | <1E-16 | | <1E-16 | | ++ | | JASPAR |
| *TROP2^c^* | NM_002353.2 | 3.56 | | | 1.11E-16 | | 7.47E-15 | | *++* | | TRANSFAC, JASPAR |
| *TGFB2* | NM_003238.2 | 2.28 | | | 4.39E-05 | | 7.74E-04 | | *+/++* | | TFS/TRANSFAC (TFTs) |
| *VCAN^c^* | NM_004385.3 | 2.03 | | | 4.36E-11 | | 1.46E-09 | | +/++ | | JASPAR |
| *B: Downregulated genes* | | | |  | | | |  | |  | |
| *ACVR1* | NM_001105.2 | -1.26 | | | <1E-16 | | <1E-16 | | +/++ | | JASPAR |
| *ACVR1C* | NM_145259.2 | -4.01 | | | 2.78E-05 | | 5.14E-04 | | +/++ | | TRANSFAC (TFTs)/JASPAR (TFTs) |
| *ADM2* | NM_001253845.1 | -1.50 | | | <1E-16 | | <1E-16 | | +/++ | | JASPAR |
| *AKT3^c^* | NM_005465.4 | -2.14 | | | 3.74E-12 | | 1.32E-10 | | +/++ | | JASPAR |
| *BTG1* | NM_001731.2 | -1.05 | | | <1E-16 | | <1E-16 | | +/++ | | JASPAR |
| *CD44^c^* | NM_001001392.1 | -1.57 | | | 1.67E-12 | | 6.20E-11 | | ++ | | ChEA |
| *CDK14* | NM_012395.2 | -3.38 | | | <1E-16 | | <1E-16 | | +/++ | | JASPAR |
| *CKMT1A* | NM_001015001.1 | -1.61 | | | 1.34E-09 | | 3.97E-08 | | ++ | | JASPAR |
| *CXCL8* | NM_000584.2 | -2.73 | | | 5.81E-07 | | 1.20E-05 | | ++ | | ENCODE (TFT) |
| *DPYSL3* | NM_001387.2 | -4.29 | | | <1E-16 | | <1E-16 | | ++ | | ChEA |
| *FHL1* | NM_001449.4 | -1.14 | | | 2.02E-13 | | 8.32E-12 | | ++ | | JASPAR |
| *FUT3* | NM_000149.3 | -7.58 | | | 2.10E-09 | | 5.97E-08 | | + | | TRANSFAC (PWM) |
| *GDF15* | NM_004864.2 | -1.54 | | | <1E-16 | | <1E-16 | | +/++ | | JASPAR |
| *HKDC1* | NM_025130.3 | -4.71 | | | <1E-16 | | <1E-16 | | ++ | | ENCODE (TFT) |
| *ID1^c^* | NM_002165.2 | -1.24 | | | 2.54E-14 | | 1.34E-12 | | +/++ | | ENCODE (ChIP-X, TFT), TRANSFAC (CTF) |
| *ISL1* | NM_002202.2 | -3.31 | | | 7.78E-13 | | 3.03E-11 | | ++ | | ENCODE (TFTs), JASPAR |
| *MCAM* | NM_006500.2 | -1.64 | | | <1E-16 | | <1E-16 | | +/++ | | JASPAR |
| *SOX9* | NM_000346.2 | -1.14 | | | 5.55E-16 | | 3.42E-14 | | +/++ | | TRANSFAC (PWM, CTF) |
| *TWIST1^c^* | NM_000474.3 | -2.29 | | | 1.07E-09 | | 3.29E-08 | | +/++ | | JASPAR |

^a^ AP-1: heterodimer of ATF2/c-JUN

^b^ Consulted databases and -sets: JASPAR Predicted Transcription Factor Targets (http://jaspar.genereg.net/), ChEA (ChIP Enrichment Analysis, https://amp.pharm.mssm.edu/Harmonizome/dataset/CHEA+Transcription+Factor+Targets), TRANSFAC (Predicted Transcription Factor using Position Weight Matrices (PWM); Curated Transcription Factor (CTF); http://genexplain.com/transfac/), ENCODE (ChIP-X, Transcription Factor Targets (TFT); https://amp.pharm.mssm.edu/Harmonizome/dataset/ENCODE+Transcription+Factor+Targets).

^c^ RT-qPCR validated (see Supplementary Figures 2H)

All gene names are in italic. *P3H2*: Prolyl 3-Hydroxylase 2; *PDGFC*: Platelet Derived Growth Factor C*; SEMA3E*: Semaphorin 3E; *SYK*: Spleen Associated Tyrosine Kinase; *TROP2*: Tumor Associated Calcium Signal Transducer 2; *TGFB2*: Transforming Growth Factor Beta 2; *VCAN*: Versican; *ACVR1*: Activin A Receptor Type 1; *ACVR1C*: Activin A Receptor Type 1C; *ADM2*: Adrenomedullin 2; *AKT3*: AKT Serine/Threonine Kinase 3; *BTG1*: B-Cell Translocation Gene 1; *CD44*: Cell Surface Glycoprotein CD44; *CDK14*: Cyclin Dependent Kinase 14; *CKMT1A*: Creatine Kinase, Mitochondrial 1A; *CXCL8*: C-X-C Motif Chemokine Ligand 8; *DPYSL3*: Dihydropyrimidinase Like 3; *FHL1*: Four And A Half LIM Domains 1; *FUT3*: Fucosyltransferase 3; *GDF15*: Growth Differentiation Factor 15; *HKDC1*: Hexokinase Domain Containing 1; ID1: Inhibitor Of DNA Binding 1; *ISL1*: ISL LIM Homeobox 1; *MCAM*: Melanoma Cell Adhesion Molecule; *SOX9*: SRY-Box 9; *TWIST1*: Twist Family BHLH Transcription Factor 1.

## Supplementary Table 5. DAVID (Database for Annotation, Visualization and Integrated Discovery) gene-annotation enrichment analysis of Gene Ontology (GO) annotated biological processes (BP) and molecular functions (MF)

| **Category** | **Biological Process** | **GO Term ID** | ***P*-value** | **Genes** |
| --- | --- | --- | --- | --- |
| GOTERM_BP_DIRECT | Endocardial cushion morphogenesis | GO:0003203 | .0001 | *ISL1, ACVR1, TWIST1, SOX9, TGFB2* |
| GOTERM_BP_DIRECT | Neural crest cell migration | GO:0001755 | .015 | *ISL1, SEMA3E, ACVR1* |
| GOTERM_BP_DIRECT | Protein phosphorylation | GO:0006468 | .024 | *ADM2, SYK, ACVR1C, ACVR1, CDK14, TGFB2* |
| GOTERM_MF_DIRECT | bHLH transcription factor binding | GO:0043425 | .028 | *ISL1, TWIST1, SOX9* |
| GOTERM_BP_DIRECT | Signal transduction by protein phosphorylation | GO:0023014 | .034 | *ACVR1C, ACVR1, TGFB2* |

GO term analysis was done using DAVID bioinformatics tool for functional annotation (*P*<0.05, Fisher’s exact test).

## Supplementary Table 6. Correlation of *TROP2* (*TACSTD2*) and *ATF2* gene expression in primary tumors (n=182) and liver metastasis (n=47).

| *A: Primary tumors* | | | | |
| --- | --- | --- | --- | --- |
| **Probes** | | ATF2-1 | | ATF2-2 |
| TACSTD2-1 | | **-0.294***** | | **-0.591***** |
| TACSTD2-2 | | -0.013 ^ns^ | | -0.082 ^ns^ |
| TACSTD2-3 | | -0.094 ^ns^ | | **-0.252***** |
| *B: Liver metastasis* | | | | |
| **Probes** | ATF2-1 | | ATF2-2 | |
| TACSTD2-1 | -0.037 ^ns^ | | **-0.526***** | |
| TACSTD2-2 | 0.060 ^ns^ | | -0.181 ^ns^ | |
| TACSTD2-3 | 0.041 ^ns^ | | **-0.553***** | |

Microarray analysis data set GSE41258; Pearson correlation, *** *P*<0.001; ns: non-significant

## Supplementary Table 7. Clinicopathological data of primary tumors and liver metastasis/regional lymph node metastasis.

|  | |  | |  | |  | |  | |  | | | **TROP2 – IHC score** | | | **ATF2 - IHC score** | | |
| --- | --- | --- | --- | --- | --- | --- | --- | --- | --- | --- | --- | --- | --- | --- | --- | --- | --- | --- |
| **No** | **Age** | | **Sex** | | **Site** | | **pT** | | **pN** | | **Stage** | **Tumor** | **Primary** | **Liver** | **Lymph node** | **Primary** | **Liver** | **Lymph node** |
|  |  | |  | |  | |  | |  | |  | **grade** | **tumor** | **metastasis** | **metastasis** | **tumor** | **metastasis** | **metastasis** |
| 1 | 61 | | male | | rectum | | 4 | | 1 | | 4 | low | 60 | 40 | 120 | 85 | 40 | 90 |
| 2 | 60 | | male | | colon | | 4 | | 2 | | 4 | high | 105 | 150 | 180 | 50 | 5 | 25 |
| 3 | 69 | | male | | colon | | 4 | | 2 | | 4 | low | 270 | 270 | n.a. | 10 | 5 | n.a. |
| 4 | 52 | | male | | colon | | 3 | | 2 | | 4 | low | 60 | 20 | 160 | 30 | 15 | 150 |
| 5 | 72 | | male | | colon | | 3 | | 1 | | 4 | low | 165 | 140 | 225 | 65 | 10 | 190 |
| 6 | 55 | | female | | colon | | 4 | | 1 | | 4 | high | 30 | 30 | 75 | 100 | 10 | 150 |
| 7 | 67 | | male | | colon | | 3 | | 1 | | 4 | high | 270 | 190 | 270 | 40 | 10 | 160 |
| 8 | 80 | | male | | colon | | 3 | | 2 | | 4 | high | 120 | 80 | 90 | 10 | 20 | 70 |
| 9 | 67 | | female | | colon | | 4 | | 2 | | 4 | high | 210 | 120 | 195 | 140 | 190 | 200 |
| 10 | 38 | | male | | colon | | 3 | | 1 | | 4 | low | 180 | 270 | n.a. | 140 | 30 | n.a. |
| 11 | 71 | | male | | colon | | 3 | | 1 | | 4 | low | 240 | 170 | 120 | 10 | 60 | 20 |
| 12 | 71 | | male | | colon | | 4 | | 0 | | 4 | high | 75 | 120 | n.a. | 90 | 60 | n.a. |
| 13 | 57 | | male | | rectum | | 3 | | 0 | | 4 | high | 205 | 60 | n.a. | 95 | 60 | n.a. |
| 14 | 50 | | male | | colon | | 3 | | 1 | | 4 | high | 220 | 270 | 270 | 80 | 20 | 60 |
| 15 | 65 | | female | | colon | | 4 | | 2 | | 4 | high | 185 | 90 | 120 | 80 | 20 | 80 |
| 16 | 66 | | female | | colon | | 2 | | 0 | | 4 | low | 140 | 10 | n.a. | 30 | 5 | n.a. |
| 17 | 67 | | female | | rectum | | 3 | | 0 | | 4 | low | 270 | 100 | n.a. | 140 | 0 | n.a. |
| 18 | 77 | | male | | colon | | 2 | | 1 | | 4 | high | 245 | 240 | 270 | 120 | 20 | 40 |
| 19 | 75 | | male | | rectum | | 2 | | 1 | | 4 | low | 170 | 125 | n.a. | 110 | 40 | n.a. |

IHC: immunohistochemistry; n.a.: lymph nodes not available

**Supplementary Table 8. Antibodies used in Western blot analysis**

| **Primary antibody (clone)** | **Cat.-No.** | **Manufacturer** | **Dilution** | **Molecular weight (kDa)** |
| --- | --- | --- | --- | --- |
| β-actin (AC-7) | A2228 | Sigma Aldrich, Germany | 1:40 000 | 42 |
| ATF2 (E242) | ab32061 | Abcam, UK | 1:10 000 | 70 |
| ATF2 (E243) | ab32160 | Abcam, UK | 1:10 000 | 70 |
| p-ATF2^Thr71^ (E268) | ab32019 | Abcam, UK | 1:5 000 | 70 |
| CRISPR/Cas9 (7A9) | C15200203 | Diagenode, Belgium | 1:1,000 | 150 |
| c-JUN (60A8) | 9165 | Cell Signaling Technologies, USA | 1:1 000 | 43/48 |
| p-c-JUN^Ser73^ (D47G9) | 3270 | Cell Signaling Technologies, USA | 1:1 000 | 48 |
| E-Cadherin | 3195 | Cell Signaling Technologies, USA | 1:2 000 | 135 |
| EpCAM | 2929 | Cell Signaling Technologies, USA | 1:2 000 | 40 |
| GAPDH (6C5) | MAB5476 | Abnova, USA | 1:100 000 | 37 |
| JNK (56G8) | 9258 | Cell Signaling Technologies, USA | 1:1 000 | 46/54 |
| p-JNK | 9251 | Cell Signaling Technologies, USA | 1:1 000 | 46/54 |
| PARP (46D11) | 9532 | Cell Signaling Technologies, USA | 1:2 000 | 89/116 |
| TROP2 (D1W5W) | 90540 | Cell Signaling Technologies, USA | 1:2 000 | 45-65 |
| **Secondary antibody** | **Cat.-No.** | **Manufacturer** | **Dilution** |  |
| Anti-biotin, HRP-linked | 7075 | Cell Signaling Technologies, USA | 1:5 000 |  |
| Goat anti-mouse IgG (H+L) | 31430 | Thermo Fischer Scientific GmbH, Germany | 1:20 000 |  |
| Goat anti-rabbit IgG (H+L) | 31460 | Thermo Fischer Scientific GmbH, Germany | 1:10 000 –1:20 000 |  |

**Supplementary Table 9. RT-qPCR primer pairs (Metabion) used in this study.**

| **Target/Purpose** | **Oligonucleotides 5’-3’** |
| --- | --- |
| *hAKT3* | *sense*: TGGAGGCCAAGATACTTCCTTT  *anti-sense*: ACTGGCATTTTGCCACTGAAAA |
| *hCD44* | *sense*: TGAATAACCTGCCGCTTTG  *anti-sense*: GCTTTCTCCATCTGGGCCAT |
| *hID1* | *sense*: AATCCGAAGTTGGAACCCCC  *anti-sense*: AGGAACGCATGCCGCC |
| *hSOX9* | *sense*: CTCTGGAGACTTCTGAACGAGAG  *anti-sense*: CCTTGAAGATGGCGTTGGGG |
| *hTROP2* | *sense*: TCCCCTTTCGGTCCAACAAC  *anti-sense*: AAACGATCCCGGGTTGTCAT |
| *hTWIST1* | *sense*: TTCTCGGTCTGGAGGATGGA  *anti-sense*: CAATGACATCTAGGTCTCCGGC |
| *hVCAN* | *sense*: GGGGTGAGAACCCTGTATCG  *anti-sense*: GATGGTTGTAGCCTCTTTAGGTTT |
| *hGAPDH* | *sense*: AGGTCGGAGTCAACGGATTT  *anti-sense*: TGGAATTTGCCATGGGTGGA |
| *hAlu* | *sense*: ACGCCTGTAATCCCAGCACTT  *anti-sense*: TCGCCCAGGCTGGAGTGCA |
| *chGAPDH* | *sense*: GAGGAAAGGTCGCCTGGTGGATCG  *anti-sense*: GGTGAGGACAAGCAGTGAGGAACG |

**Supplementary Table 10. ChIP primer pairs (Metabion) used in this study.**

| **Target/Purpose** | **Oligonucleotides 5’-3’** |
| --- | --- |
| *TROP2* site 1 | *sense*: CCTGTTCTGATCCTATCGCGG  *anti-sense*: TATACTCTACCCGACCTGCCC |
| *TROP2* site 2 | *sense*: AACTGCTTCCCTAACTTCCCTTC  *anti-sense*: AGACCCGTTTTATAGGTGGTACT |
| *TROP2* site 3 | *sense*: GCCTGTAATCCCAGCTACTC  *anti-sense*: GAAGCAGAGACATGTGAGGGA |
| *TROP2* site 4 | *sense*: TAGAACCTGACTCAGATATCTGTC  *anti-sense*: ATTGTGGGATAGTCCTCTGCTG |
| *TROP2* site 5 | *sense*: CAGGGAGGGCCTTATTAGGGAA  *anti-sense*: CCTACCTGGAGTCTCCCTTTCTT |

**Supplementary Table 11. Antibodies used for immunohistochemistry.**

| **Primary antibody (clone)** | **Cat.-No.** | **Manufacturer** | **Dilution** |
| --- | --- | --- | --- |
| Mouse monoclonal anti-Ki67 (Mib-1)^a^ | M7240 | Dako/Agilent | 1:100 |
| Rabbit monoclonal anti-ATF2 (20F1)^b^ | 9226 | Cell Signaling Technologies | 1:50^c^ |
| Rabbit monoclonal anti-ATF2 (E243)^d^ | ab32160 | Abcam | 1:10 000^f^  1:20 000^g^  1:100 000^h^ |
| Rabbit monoclonal anti-TROP2 (EPR20043)^e^ | ab214488 | Abcam | 1:2 000 |
| **Secondary antibody** | **Cat.-No.** | **Manufacturer** | **Dilution** |
| Goat polyclonal anti-rabbit (biotinylated) | BA-1000 | Vector Laboratories | 1:100 |

^a^ Immunohistochemical staining performed in the clinical routine setting on a Ventana BenchMark Ultra automated instrument (Ventana Medical Systems, Inc.), combined with UltraView Universal DAB Detection Kit (includes secondary antibodies; Roche).

^b^ heat-induced antigen-retrieval in TRS buffer, pH9

^c^ for human whole tissue sections

^d^ heat-induced antigen-retrieval in TRS buffer, pH 6

^e^ heat-induced antigen-retrieval in citrate buffer, pH6

^f^ dilution used for human TMA sections

^g^ dilution used for CAM sections

^h^ dilution used for all other CAM/mouse sections

# Supplementary Material and Methods

## Human CRC cohort

This study was covered by ethic votes of the University Hospital of the Friedrich-Alexander University Erlangen-Nürnberg (24.01.2005, 18.01.2012). All performed procedures involving human participants were in accordance with the ethical standards of the institutional and/or national research committee and with the 1964 Helsinki declaration and its later amendments or comparable ethical standards.

We retrospectively analyzed a cohort of 332 patients with primary adenocarcinoma of the colon who underwent surgical resection at the University Hospital Erlangen (2005 and 2009). Patients having received neoadjuvant therapy before surgery were excluded. Clinical data and follow-up information were collected by the Erlangen Registry for Colorectal Carcinoma (ERCRC). Detailed information for this patient cohort is given in Supplementary Table 1. Histopathological review of all diagnostic cases was performed independently by three pathologists and tumors were staged according to the eighth edition of the UICC TNM classification.

Another cohort comprised 19 patients with primary adenocarcinoma of the colon and its corresponding liver metastasis who underwent surgical resection at the University Hospital Erlangen. For 12 of 19 patients, tissue blocks of lymph node metastasis were available. Clinical data were collected by the Erlangen Registry for Colorectal Carcinoma (ERCRC) and are given in Supplementary Table 7. Histopathological review was performed as stated above.

Tissue microarrays (TMAs) were constructed as previously described.[[1](#_ENREF_1), [2](#_ENREF_2)] The presence of complete clinical datasets of each patient was required for clinical correlation analysis. Tumor heterogeneity was considered by evaluating 2-3 punches from different tumor regions. Therefore, we excluded cases in which only one punch could be analyzed to decrease experimental bias.

Immunoscores were determined by multiplying staining intensity (0-3) with the corresponding percentage of positively stained tumor cells (0-100%). Heatmap illustrations were generated using the “gradient map visualization module” of the 3DHistech Case Viewer software.

## Cell culture

HCT116 (ATCC^®^ CCL-247) and HT29 (ATCC^®^ HTB-38) cells as well as the corresponding ATF2- and TROP2-KO clones were cultured in RPMI 1640 medium (PAN Biotech, Aidenbach, Germany), supplemented with 10% fetal bovine serum (FBS, PAN Biotech) and 1% penicillin/streptomycin (P/S, PAN Biotech). HEK293 cells (ATCC^®^ CRL-1573™) were cultured in DMEM (Sigma-Aldrich, St. Louis, MO, USA) supplemented with 10% FBS (Thermo Fisher, Waltham, MA, USA) and 1% P/S (Biosera, Nuaille, France). All cell lines were obtained from ATCC and regularly tested negative for mycoplasma contamination. HCT116 and HT29 cells (wildtype, ATF2-KO, TROP2-KO) were authenticated using Multiplex Cell Authentication by Multiplexion (Heidelberg, Germany), as described recently.[[3](#_ENREF_3)]

## Generation of stable ATF2-knockout (KO) cells

*ATF2*-knockout (KO) cells were established in the colorectal cancer cell lines HCT116 and HT29 using the CRISPR/Cas9 technique as previously described with minor modifications.[[4](#_ENREF_4)] Single guide RNA (sgRNA; Supplementary Figure 2A,B) targeting exon 5 of human *ATF2* (NM_001880.4; ENSG 00000115966) was designed using Benchling (<https://benchling.com>) and cloned into pX330-U6-Chimeric_BB-CBh-hSpCas9 (Addgene plasmid #42230) [[5](#_ENREF_5)] at its *Bbs*l restriction site (= pX330-ATF2-sgRNA). HCT116 (0.7×10^6^) and HT29 (0.6×10^6^) cells were seeded into 6-well plates and co-transfected with 1.25 µg of pX330-ATF2-sgRNA and 1.25 µg of pBABE-Puro (Addgene plasmid #1764) [[6](#_ENREF_6)] using Lipofectamine 2000 (Life Technologies, Carlsbad, CA, USA) according to the manufacturer’s protocol. At 24 h post-transfection, cells were treated with 1.5–2.5 µg/ml puromycin for up to 20 days to select for stably transfected cells which were subsequently plated in limiting dilutions for monoclonalization. ATF2-KO was evaluated by Western blot and Sanger sequencing (Seqlab, Goettingen, Germany). For sequencing, the region of interest was amplified prior to sequencing using the following set of primers: sense: 5’-TGCCACAGCGTTTTACCAAC-3’, anti-sense:
5’-CACAGCCTTGTAAGTCAGTTGT-3’. The sense primer was used for Sanger sequencing. Parental HCT116 ATF2-WT and HT29 ATF2-KO cells are hereafter referred to as HCT116 and HT29 cells, respectively.

## Generation of stable TROP2-knockout (KO) cells

TROP2-knockout (KO) cells were generated by using CRISPR/Cas9 in HCT116 and HT29 wildtype cells as well as in the ATF2-KO clones F9 (HCT116) and F10 (HT29). For targeting the TROP2 gene *TACSTD2*, cells were transfected with a CRISPR plasmid (vector ID: VB900048-8111hzp, pRP[CRISPR]-EGFP/Puro-hCas9-U6>hTACSTD2[gRNA#179]) expressing Cas9 and the guide sequence targeting *TACSTD2* (guide sequence: 5’-GGCGTGCGCCGCACGGACAA-3’). For this, HCT116 (0.4×10^6^) and HT29 (0.8×10^6^) wildtype cells and ATF2-KO clones were seeded into 6-well plates and transfected with 2.5 µg of the CRISPR/Cas9 plasmid using Lipofectamine 2000 (Life Technologies, Carlsbad, CA, USA) according to the manufacturer’s protocol. At 24 h post-transfection, cells were treated with
2.0 µg/ml (HCT116, F9) and 2.5 µg/ml (HT29, F10) puromycin for 5 days to select for stably transfected cells which were subsequently plated in limiting dilutions to generate clones of monoclonal origin. TROP2-KO was evaluated by Western blot and Sanger sequencing (Seqlab, Goettingen, Germany). Prior sequencing, the region of interest was amplified using the following set of primers: sense: 5’-CAAGGCGCGCCAGTGCAACC-3’, anti-sense: 5’-CGACTTTCTCCGGTTGGTGA-3’. The sense primer was used for Sanger sequencing.

## NanoString gene expression analysis

Gene expression analysis was performed using the human nCounter^®^ PanCancer Progression Panel (NanoString Technologies, Seattle, WA, USA) according to the manufacturer’s protocol with 100 ng of total RNA from HCT116, F9, and E5 cells. Analysis of NanoString expression data was performed relying on the package NanoStringDiff v. 1.14.0 [[7](#_ENREF_7)] within an R v. 3.6.1/Bioconductor v. 3.9 environment.[[8](#_ENREF_8), [9](#_ENREF_9)] For visualization purposes, normalized expression values were log2-transformed (after addition of an offset equal to 1). NanoStringDiff data normalization was used to adjust for positive and housekeeping size factors as well as background noise. A heatmap for selected differentially expressed genes (adjusted *P*<0.01, absolute (log2(fold change, FC))>1 in the comparison of both HCT116 ATF2-KO clones versus parental HCT116 cells was obtained relying on gplots v. 3.0.1.1.[[10](#_ENREF_10)] To this aim, log2-transformed normalized expression values were standardized. A volcano plot was produced relying on EnhancedVolcano v.1.2.0.[[11](#_ENREF_11)] In this plot, only genes with absolute (log2(FC))<10 are shown and null adjusted p-values returned by NanoStringDiff have been replaced by 1E-16. Functional enrichment analysis of the 26 selected genes was performed using the Functional Annotation Tool of DAVID bioinformatics resources v. 6.8[[12](#_ENREF_12), [13](#_ENREF_13)] using the 740 panel genes as background and GOTERM_BP_DIRECT and GOTERM_MF_DIRECT as categories. The newly identified ATF2-mediated gene signature and potential protein-protein interactions were evaluated using the STRING interaction database[[14](#_ENREF_14)] (<https://string-db.org>, version 11.0).

## QuPath analysis

QuPath analysis was performed on 20 randomly selected whole tissue sections out of the CRC cohort (n=332). ATF2 and TROP2-stained whole slides were scanned on a slide scanner (P250 Flash, 3DHistech, Hungary) and tumor tissue was annotated by two pathologists (KEW, ME) in a standardized manner and fully quantitatively analyzed using the open source software QuPath 0.2.3 (<https://qupath.github.io>).[[15](#_ENREF_15)] The AI-based H-score is a parameter combining the percentage of positively stained cells with the staining intensity levels (0 = none, 1 = weak, 2 = moderate, 3 = strong). Thus, a maximum score of 300 can be reached. Staining was quality controlled for presence of tumor tissue and proper staining results. Tissue areas with staining artefacts (e.g. DAB shades) were excluded from the analysis.

## Bioinformatics analysis

Raw expression and clinical data for 90 (metastatic: n=25, non-metastatic: n=65) primary tumor samples from GSE2109, were downloaded from the Gene Expression Omnibus (GEO) database using *GEOquery* package. GSE2109 was processed automatically using its default package. Expression signals were normalized using robust multi-array average (RMA) expression measure from *affy[*[*16*](#_ENREF_16)*,* [*17*](#_ENREF_17)*]* and the respective probes of each gene were extracted from annotation files. For GSE2109, probes 227128_s_at (TROP2), 205446_s_at (Probe 1) and 212984_s_at (Probe 2; ATF2) were used for further analysis.

Pre-aligned, raw HTSeq format RNA sequencing (RNASeq) read counts and their accompanying clinical data for the colon adenocarcinoma (COAD) cohort were downloaded using *TCGABiolinks* package.[[18](#_ENREF_18), [19](#_ENREF_19)] After the dataset was cleaned, the read counts were normalized by utilizing the DESeq2 package[[20](#_ENREF_20)] and the read counts were transformed according to the variance stabilizing transformation (VST) method to avoid bias introduced by the conventional log2(expression + constant) transformation.[[21](#_ENREF_21)] Forty-one tumors were matched to their adjacent normal tissues by patient unique ID and differential expression analysis was performed with *DESeq2* package using a general linear model (GLM) for sample status (tumor vs. normal) and sequencing method (hiseq vs genome analyzer). Statistical significance was determined by Wald Test’s statistic adjusted for multiple hypothesis testing according to Benjamini and Hochberg’s procedure (FDR).[[20](#_ENREF_20)]

We used overall survival (OS) from the COAD dataset to determine the clinical relevance of *ATF2* and *TROP2* genes individually. In addition, we designed a combined score of ATF2-*TROP2* gene expression to determine their combined effect on patient survival. To get the optimal patient separation according to either individual gene or combined gene score, we used the *surv_cutpoint()* function from the *survminer* package. Log-rank p-values and hazard ratios were calculated using the relevant functions from the *survival* package. All these analyses were performed using R programming language, version 4.1.0, on a Linux operating system.

Raw expression data and their accompanying clinical information from GSE41258 dataset [[22](#_ENREF_22)] using primary tumors (PT, n=182) of the colon and colon metastasis into the liver (n=47) were downloaded from Gene Expression Omnibus (GEO) database. This dataset comprises three different TACSTD2 probes and two different ATF2 probes. Expression signals for all samples were normalized simultaneously using robust multi-array average (RMA) expression measure from *affy [*[*16*](#_ENREF_16)*,* [*17*](#_ENREF_17)*]* and probes were annotated using the respective package. Probes of the genes of interest were extracted and used for further analysis.

## Chromatin immunoprecipitation

Chromatin immunoprecipitation (ChIP) was performed using the ChIP-IT High Sensitivity Kit (Active Motif, Carlsbad, CA, USA) according to the manufacturer’s protocol. For this, 2.5×10^6^ HCT116 cells (wildtype and ATF2-KO) and 3.0×10^6^ HT29 cells (wildtype and ATF2-KO) were seeded on 15 cm plates and incubated for 72 h. For JNK inhibition, HCT116 and HT29 cells were treated with either 10 µM SP600125 or an equal amount of DMSO at 48 h post-seeding for additional 24 h. Then, cells were collected, fixed for 15 min, and lysed using ready-to-use buffers supplied by the kit. Chromatin was sonicated, and successful shearing was validated by agarose gel electrophoresis. Chromatin (30 µg) was immunoprecipitated with 5 µg anti-ATF2 antibody (ab32160; Abcam, Cambridge, UK), 4.5 µg anti-c-JUN (Abcam, ab31419), and with the corresponding amounts of rabbit IgG antibody (#3900; Cell Signaling, Danvers, MA, USA) at 4 °C overnight. For c-JUN, ChIP of HCT116 and ATF2-KO clones, amounts of chromatin and antibody were scaled up (100 µg chromatin, 15 µg anti-c-JUN/rabbit IgG antibody). After pull down with agarose G beads, eluted ChIP DNA was de-cross-linked by Proteinase K digestion and purified. RT-qPCR was performed with the eluted ChIP DNA using specific primers for putative binding sites in the *TROP2* promoter. Fold enrichment of the targeted sequences was calculated over IgG (fold enrichment = 2^-(Ct^_IP_^− Ct^_IgG_^)^) as previously described.[[23](#_ENREF_23)] All ChIP-qPCR runs were quality-checked by melt curve analysis. Primers used for ChIP-qPCR are given in Supplementary Table 8.

## Chorioallantoic membrane (CAM) assay

Fertilized specific-pathogen-free eggs (VALO BioMedia; LSL Rhein-Main) were used and cultured at 37 °C in a humidified atmosphere. Eggs were opened at embryonic developmental day 8 (EDD 8) and sealed with tape. On EDD 9, 1.0×10^6^ cells (if not stated otherwise in the figure legends) were resuspended in a 1:1 medium/Matrigel (Corning) mixture and droplets of the cell/Matrigel suspension were formed and cured. One pellet per egg was grafted onto the CAM. After five days of incubation, the developed ovografts were resected, documented *ex ovo,* and fixed in 4% formaldehyde for 24 h for the generation of formalin-fixed paraffin-embedded blocks. Only eggs with a complete set of markers were evaluated for IHC analysis, scoring, and Alu qPCR. Based on the official gazette of the European Union, the CAM assay is considered as a non-animal experiment until hatching and contributes to the 3R principles.[[24](#_ENREF_24)]

## Detection of disseminating tumor cells by Alu qPCR

Analysis of the dissemination potential of HCT116 and HT29 cells upon ATF2 loss was determined by Alu qPCR in organs of the chicken embryo based on the CAM assay, as previously described.[[25](#_ENREF_25)] Briefly, for Alu qPCR, chicken livers were harvested at day five post-engraftment and dissociated, and genomic DNA (gDNA) was isolated. Human Alu sequences within the isolated gDNA samples (200 ng) were amplified using specific primers (Supplementary Table 7) and the QuantiTect SYBR^®^ Green PCR Kit (Qiagen) according to the manufacturer’s protocol. Quantification of human Alu sequences was carried out with a CFX96^TM^ Real-Time System (Bio-Rad) and the C1000^TM^ Thermal Cycler (Bio-Rad). Obtained values were normalized to chicken GAPDH, and relative human Alu sequence levels were determined with regard to a human gDNA control sample (Human Genomic DNA, Human Mixed, 100 µg, G3041, Promega). Human gDNA of 0.01 ng was defined as a relative value of 1.0. The cut-off for metastasis detection was set at 0.5 and, thus, relative values <0.5 were defined as having no existent metastasis.

## RNA interference

For RNA interference mediated gene silencing, 3.5×10^5^ cells were seeded into 6-well plates. Cells were transfected with either *TROP2*-specific (si) ON-TARGETplus siRNA or non-targeting control (scr) SMARTpools (Dharmacon, Lafayette, CO, USA) for 48 h using Lipofectamine RNAiMAX according to the manufacturer’s protocol (final: 25 pmol/well of siRNA/scr). Knockdown efficiency was assessed by RT-qPCR.

## RT-qPCR

Total RNA from cell pellets was extracted using QIAzol^®^ Lysis Reagent (Qiagen) combined with RNeasy Mini Kit (Qiagen) according to the manufacturer’s instructions. Reverse transcription was performed with 1 µg of total RNA using the QuantiTect Reverse Transcription Kit (Qiagen, Hilden, Germany). cDNA amplification was conducted using primers for targets of interest (Metabion; Supplementary Table 7) and QuantiTect or QuantiFAST SYBR^®^ Green PCR Kit (Qiagen) according to the manufacturer’s protocol. Ct expression values were determined using a CFX96^TM^ Real-Time System (Bio-Rad) and a C1000^TM^ Thermal Cycler (Bio-Rad, Hercules, CA, USA). Obtained Ct values were normalized to human GAPDH expression.

## Western blot

Cells pellets were collected and lysed, and western blotting was performed as previously described.[[4](#_ENREF_4), [26](#_ENREF_26)] Briefly, proteins (30-40 µg) were separated by SDS-PAGE and transferred onto nitrocellulose membranes (GE Healthcare, Chicago, IL, USA). After blocking for unspecific binding, membranes were incubated with primary antibodies (Supplementary Table 6) at 4 °C overnight. Membranes were then probed with HRP-conjugated secondary antibodies (Supplementary Table 6) for 1 h at RT. Signals were detected using the Immobilon Western Chemiluminescent HRP substrate kit (Merck Millipore, Burlington, MA, USA) and the GeneGnome imaging system (Syngene, Bangalore, India).

## Wound healing migration assay

Cells of HT29 and ATF2-KO clone B5 were transfected with *TROP2*-specific (si) or non-targeting (scr) RNAi for 48 h as described in the Methods section “RNA interference”. At 48 h post-transfection, 1.2×10^5^ cells were re-seeded into each well of a 2-well culture insert (Ibidi, Graefelfing, Germany) mounted on 12-well plates. To assess proliferation-independent effects, cells were treated with 1.0 µg/ml mitomycin C (Sigma Aldrich) on the next day, 2 h prior to removal of the culture insert. Upon elimination of the culture insert resulting in a defined cell-free gap, cells were further cultured in medium containing 1.0 µg/ml mitomycin C. Images of the gap area were taken at 0 h, 24 h, and 48 h after insert removal by light microscopy at 4x magnification (Leica DMi1, Leica Microsystems, Wetzlar, Germany). The gap area was annotated and measured using ImageJ (National Institutes of Health, Bethesda, MD, USA).

## 3D tumor spheroid invasion and migration assays

Spheroids were generated as previously described[[4](#_ENREF_4)] with some minor modifications. Briefly, for both assays, 1.0×10^3^ cells were seeded into 96-well ultra-low attachment (ULA) round bottom plates (Corning^®^ Inc., Corning, NY, USA) and spheroids were allowed to grow for seven days at 37 °C with 5% CO_2_ in a humidified atmosphere.

A spheroid invasion assay was performed as previously described.[[4](#_ENREF_4)] Spheroid invasion was documented by light microscopy at 4× magnification (Leica DMi1, Leica Microsystems) every 24 h. Invasion areas were manually annotated using GIMP (GNU Image Manipulation Program, version 2.8) and Photoshop CS5 (Adobe, San Jose, CA, USA), and images were processed using ImageJ 1.46r (National Institutes of Health) and a self-written macro (see Supplementary Materials).

Spheroid migration was assessed as previously described[[4](#_ENREF_4)] with some minor adaptations. Generated spheroids were transferred into flat-bottom 24-well plates (one spheroid per well) containing 500 µl culture medium and incubated for up to 72 h. Spheroid migration was documented by light microscopy at 4× magnification (Leica DMi1, Leica Microsystems) every 24 h after transfer (t=0 h). Migration areas were manually annotated using GIMP (GNU Image Manipulation Program, version 2.8) and Photoshop CS5 (Adobe), and images were processed using ImageJ 1.46r (National Institutes of Health) and a self-written macro (see Supplementary Materials).

## Immunofluorescence

For immunofluorescence, 4.0×10^5^ cells were seeded onto fibronectin-coated coverslips and allowed to adhere overnight. Then, cells were fixed in 4% PFA, permeabilized in 0.1% Triton X-100 (in PBS), and blocked in 1% BSA solution. Cells were incubated with the primary antibodies rabbit anti-TROP2 (1:500, EPR20043; Abcam, ab214488) or mouse anti-Paxillin (1:100, clone 349; BD Biosciences, 610051) for 1 h at RT and subsequently probed with secondary antibodies anti-rabbit IgG (H+L) Alexa Fluor 488 (1:500, A-11034; Invitrogen, Waltham, MA, USA) or anti-mouse IgG Alexa Fluor 488 (1:500, Invitrogen), respectively, for 30 min at RT. The cytoskeleton was visualized by phalloidin staining (1:40, #PHDH1-A, Cytoskeleton Inc., Denver, CO, USA) for 20 min at RT. Nuclei were visualized with DAPI (1:1000; Sigma Aldrich; #MBD0015). Coverslips were mounted in Fluoromount medium (Sigma Aldrich, F4680) and imaging was performed on Nikon Eclipse Ti-S at 100× magnification or on a Zeiss LSM710 at 63× magnification.

## Quantification of filopodia

Filopodia orientation was manually quantified upon visual inspection. For this, the number of cells with directed and undirected filopodia orientation on each image was counted and evaluated by the following criteria. First, only cells that were clearly visible and not cut-off by image borders were categorized into one of three groups: no filopodia, directed filopodia, and undirected filopodia. Cells without any filopodia at the outer membrane were categorized into the “no filopodia” group. In contrast, cells with filopodia between cells or directed towards adjacent cells were counted as “directed filopodia”. Only if cells with filopodia, which did not show any directionality towards attached or adjacent cells (independently of any other directed filopodia), these cells were counted as cells with “undirected filopodia”. Evaluation criteria for filopodia quantification are given in Supplementary Figure 4B.

## Immunohistochemical staining and analysis

Formalin-fixed and paraffin-embedded sections (1-3 µm) of human TMA, CAM, or mouse origin were deparaffinized with xylene and rehydrated with graded ethanol according to standard procedures. Validated immunohistochemical (IHC) protocols established at our institute for clinical routine were applied for haematoxylin and eosin (HE) and IHC staining for Ki67 (Mib-1, Dako/Agilent, M7240). IHC was performed on a Ventana BenchMark Ultra automated instrument (Ventana Medical Systems, Inc., Oro Valley, AZ, USA) in combination with the UltraView Universal DAB Detection Kit (Roche, Basel, Switzerland) according to the manufacturer’s recommendations.

For ATF2 (Cell Signaling, Abcam) and TROP2 (Abcam) IHC staining, heat-induced antigen retrieval was followed by blocking of endogenous peroxidase (Dako, Santa Clara, CA, USA) prior to incubation with primary antibodies overnight at RT. Sections were further incubated with biotinylated secondary antibody goat anti-rabbit (Vector Laboratories, Burlingame, CA, USA). Immunoreactions were detected using VECTASTAIN^®^ Elite^®^ ABC Kit (Vector Laboratories) and DAB substrate (Dako) and sections were counterstained with haematoxylin. Antibodies used in this study are listed in Supplementary Table 9.

Stained sections were scanned using either a Pannoramic MIDI system (Camera type: CIS VCC-FC60FR19CL; objective: Plan-Apochromat; magnification: 40x; Camera adapter magnification: ×1, 3DHISTECH) or a Pannoramic 1000 system (Camera type: Adimec Q-12A-180Fc; objective: Plan-Apochromat; objective magnification: 20x; Camera adapter magnification: ×1.6; 3D-Histech) for digital analysis. Scoring and image analysis was performed using CaseViewer software (3DHISTECH). Immunoreactivity was either analyzed by determining the percentage of positively stained cells (CAM, xenografts) or by assessing the ATF2 score in a semi-quantitative manner (human TMA).

## Lentiviral vector preparation and cell transduction for luciferase-labelled cell lines

To prepare 10 ml of lentiviral supernatant, HEK293 cells seeded in a 10 cm dish at 90% confluence were transfected with pHIV-iRFP720-Luc dual reporter vector[[27](#_ENREF_27)] (8.44 μg, Addgene plasmid #104587) together with packaging plasmid pCMV-dR8.2 dvpr[[28](#_ENREF_28)] (7.55 μg; Addgene plasmid #8455) and envelope plasmid pCMV-VSV-G[[28](#_ENREF_28)] (0.88 μg; Addgene plasmid #8454) using polyethylenimine (PEI, 50 μg, Polysciences). The viral medium was harvested after 72 h of transfection and filtered with 0.45 μm PVDF filter. For *in vivo* applications, HCT116 and ATF2-KO cells were transduced by mixing their culture medium with lentiviral supernatant at a 1:1 ratio and incubated overnight at 37 °C.

## Subcutaneous murine xenograft model and IHC analysis

Mouse experiments were conducted in accordance with institutional guidelines of the Institute of Molecular Genetics, Czech Academy of Science, and approved under the project license PP63-2018. Thirteen- to fifteen-week old, male, immunodeficient mice (NOD.Cg-Prkdc^scid^ Il2rg^tm1Wjl^/SzJ) were purchased from The Jackson Laboratory[[29](#_ENREF_29), [30](#_ENREF_30)] and housed under specific-pathogen-free conditions with daily 12 h light and 12 h dark cycles.

Tumors were generated by subcutaneous injection of 0.5×10^6^ luciferase-labelled HCT116 and ATF2-KO cells in a 1:1 dilution of PBS:Matrigel (Corning® Matrigel®, #356230) into the flanks of immunodeficient mice using a 26G needle (n≥7 per group). Tumor growth was regularly monitored by bioluminescence imaging (BLI) starting one week after injection to determine tumor size and animal welfare. Mice were imaged twice a week for at least 28 days and were then sacrificed (if not earlier due to animal welfare ethics) for resection of developed tumors. BLI was performed using the Xtreme In Vivo Imaging system (Bruker, Billerica, MA, USA) to detect emitted signals. Animals were anesthetized by intramuscular injection of 50 µl of 20% zoletil (from stock solution of the mixture of 25 mg/kg of zoletil 100 (Biopharm) and 10 mg/kg of xylazin (Rometar, Biopharm) and received an intraperitoneal injection of D-Luciferin (XenoLight D-Luciferin 15 mg/ml; Perkin Elmer, Waltham, MA, USA) at a dose of 165 mg/kg. Images were taken and processed using Molecular Imaging software (Bruker). Resected tumors were fixed for 10 days in 4% PFA for further histological analysis. Each tumor was segmented to obtain 2-3 paraffin blocks that were serially cut, and HE slides of three levels per block were analyzed to score the overall depth of tumor cell invasion into the muscle layer of each animal.

## Micro-CT imaging

Micro-CT imaging was used for the detailed study of possible muscle layer tumor invasion upon subcutaneous xenograft formation. Three (HCT116, E5) to four (F9) weeks after injection, tumors were excised with surrounding muscle tissue and fixed with 4% PFA for 10 days. Tumors were stained with 25% Lugol’s solution (I_2_ and KI in aqueous solution) for two weeks and embedded in 2.5% SeaKem^®^ LE Agarose prior to imaging. Micro-CT imaging of stained and embedded xenografts was performed using a SkyScan 1176 micro-CT system (Bruker) with the following parameters: image pixel size 9 µm, filter Al 1 mm, detector binning 1×1, 360° scan, exposure time 2000 ms, rotation step 0.29, source voltage (kV)=50, source current (µA)=370. Subsequently, raw micro-CT data were reconstructed using InstaRec Bruker software (ring artefact correction 7, smoothing 1, beam hardening correction 34 %). Three-dimensional datasets were segmented to discriminate between tumor and muscle tissue using CTAn software (Bruker, according to tissue density) and, finally, these projections were visualized using CTVox software (Bruker).

## Statistical Analysis

Statistical analyses were performed with GraphPad Prism v. 8.3.0 (GraphPad, San Diego, CA, USA) and IBM® SPSS® Statistics software package v. 24.0 (IBM, USA). Statistical testing, N numbers and *P*-values for statistical significance are described in the figure legends. Statistical significance was defined as *P*<0.05.

## ImageJ macros

**1. Anchorage independent growth assay**

//This function renames the ROIs in a numerical order starting from 1.

function setNameROI(){

NrROI = roiManager("count");

for (i = 0; i < NrROI; i ++1) {

//roiManager("deselect");

roiManager("select", i);

roiManager("rename", i+1);

}

};

//This function saves the ROIs in the ROI manager to a zip file in a separate folder called "ROIs" in the chose output folder.

function saveROIs(outputFolder){

filename = getInfo("image.filename");

lenFilename = lengthOf(getInfo("image.filename"));

newEnd = substring(filename,0, lenFilename - 4);

outputPath = outputFolder + "/Results/ROIs/" + newEnd + ".zip";

roiManager("save", outputPath);

};

//This function measures the detected ROIs and saves them as a text file

function measureROI(outputFolder){

//Sets the measurements that are recorded for each slice

run("Set Measurements...", "area display redirect=None decimal=3");

roiManager("deselect");

run("Clear Results");

roiManager("measure");

filename = getInfo("image.filename");

lenFilename = lengthOf(filename);

newEnd = substring(filename,0, lenFilename - 4);

outputPath = outputFolder + "/Results/" + newEnd + ".txt";

selectWindow("Results");

saveAs("Text",outputPath);

//roiManager("deselect");

roiManager("delete");

};

//This function saves the pictures with overlays as a JPEG.

function saveOverlay(outputFolder, filename) {

outputPath = outputFolder + filename;

//saves as file as jpg

saveAs("Jpeg", outputPath);

};

//With this function the ROIs are generated.

function count(inputFolder,filename) {

open(inputFolder + filename);

//sets scale to predetermined values from calibration slide

run("Set Scale...", "distance=665 known=1000 pixel=1 unit=µm global");

run("32-bit");

//Segmentation

setAutoThreshold("Triangle");

setOption("BlackBackground", false);

run("Convert to Mask");

run("Maximum...", "radius=.5");

run("Fill Holes");

run("Watershed");

run("Analyze Particles...", "size=950-Infinity show=[Overlay Outlines] display exclude clear include add summarize");

if (Overlay.size > 0) {

//run("To ROI Manager");

close();

//Reopens the original image and pastes the outlines of the determined particles onto it

open(inputFolder + filename);

run("From ROI Manager");

}

else {

close();

call("java.lang.System.gc");

}

};

//Start of the script:

call("java.lang.System.gc");

run("Clear Results");

inputFolder = getDirectory("Choose the input folder!");

outputFolder = getDirectory("Choose the output folder!");

File.makeDirectory(outputFolder + "/Results/");

File.makeDirectory(outputFolder + "/Results/"+"/ROIs/");

//batchmode will increase processing speed. For test routines it should be set on false

setBatchMode(true);

imageList = getFileList(inputFolder);

//iteration that runs through all images

for (i=0; i<imageList.length; i++) {

count(inputFolder,imageList[i]);

setNameROI();

saveROIs(outputFolder);

saveOverlay(outputFolder,imageList[i]);

measureROI(outputFolder);

//run("Clear Results");

close();

}

selectWindow("Results");

run("Close");

selectWindow("Summary");

saveAs("Text",outputFolder + "Summary.txt");

run("Close");

//selectWindow("ROI Manager");

//run("Close");

**2. Annotation of spheroid areas of 3D migration assay**

function SpheroidArea(inputFolder,filename) {

open(inputFolder + filename);

//sets scale to predetermined values from calibration slide

run("Set Scale...", "distance=665 known=1000 pixel=1 unit=µm global"); //Leica microscope in cell culture 4x magnification

run("Channels Tool... ");

run("Make Composite");

Stack.setDisplayMode("grayscale");

Stack.setChannel(2);

setAutoThreshold("Default dark");

//run("Threshold...");

setThreshold(0, 90);

run("Convert to Mask", " ");

Stack.setChannel(3);

//run("Threshold...");

setAutoThreshold("Default dark");

setThreshold(0, 255);

run("Convert to Mask", " ");

Stack.setChannel(2);

run("Analyze Particles...", "size=100-Infinity pixel circularity=0.00-1.00 show=Nothing display slice");

Stack.setChannel(3);

run("Analyze Particles...", "size=100-Infinity pixel circularity=0.00-1.00 show=Nothing display slice");

};

//Start of the script:

call("java.lang.System.gc");

run("Clear Results");

inputFolder = getDirectory("Choose the input folder!");

//batchmode will increase processing speed. For test routines it should be set on false

setBatchMode(true);

imageList = getFileList(inputFolder);

//iteration that runs through all images

for (i=0; i<imageList.length; i++) {

SpheroidArea(inputFolder,imageList[i]);

close();

}

selectWindow("Results");

saveAs("Measurements", "" + inputFolder + "Results.txt");

run("Close");

# Supplementary References

1 Böhm J, Muenzner JK, Caliskan A, Ndreshkjana B, Erlenbach-Wunsch K, Merkel S *et al*. Loss of enhancer of zeste homologue 2 (EZH2) at tumor invasion front is correlated with higher aggressiveness in colorectal cancer cells. *J Cancer Res Clin Oncol* 2019; 145: 2227-2240.

2 Nolte S, Zlobec I, Lugli A, Hohenberger W, Croner R, Merkel S *et al*. Construction and analysis of tissue microarrays in the era of digital pathology: a pilot study targeting CDX1 and CDX2 in a colon cancer cohort of 612 patients. *J Pathol Clin Res* 2017; 3: 58-70.

3 Castro F, Dirks WG, Fähnrich S, Hotz-Wagenblatt A, Pawlita M, Schmitt M. High-throughput SNP-based authentication of human cell lines. *Int J Cancer* 2013; 132: 308-314.

4 Steinmann S, Kunze P, Hampel C, Eckstein M, Bertram Bramsen J, Muenzner JK *et al*. DAPK1 loss triggers tumor invasion in colorectal tumor cells. *Cell Death Dis* 2019; 10: 895.

5 Cong L, Ran FA, Cox D, Lin S, Barretto R, Habib N *et al*. Multiplex genome engineering using CRISPR/Cas systems. *Science* 2013; 339: 819-823.

6 Morgenstern JP, Land H. Advanced mammalian gene transfer: high titre retroviral vectors with multiple drug selection markers and a complementary helper-free packaging cell line. *Nucleic acids research* 1990; 18: 3587-3596.

7 Wang H, Horbinski C, Wu H, Liu Y, Sheng S, Liu J *et al*. NanoStringDiff: a novel statistical method for differential expression analysis based on NanoString nCounter data. *Nucleic acids research* 2016; 44: e151.

8 R Core Team (2019). R: A language and environment for statistical computing. R Foundation for Statistical Computing, Vienna, Austria.

9 Huber W, Carey VJ, Gentleman R, Anders S, Carlson M, Carvalho BS *et al*. Orchestrating high-throughput genomic analysis with Bioconductor. *Nat Methods* 2015; 12: 115-121.

10 Warnes GR, Bolker B, Bonebakker L, Gentleman R, Huber W, Liaw A *et al*. gplots: Various R Programming Tools for Plotting Data. R package version 3.0.1.1., 2019.

11 Blighe K. EnhancedVolcano: Publication-ready volcano plots with enhanced colouring and labeling. R package version 1.2.0., 2019.

12 Huang da W, Sherman BT, Lempicki RA. Systematic and integrative analysis of large gene lists using DAVID bioinformatics resources. *Nat Protoc* 2009; 4: 44-57.

13 Huang da W, Sherman BT, Lempicki RA. Bioinformatics enrichment tools: paths toward the comprehensive functional analysis of large gene lists. *Nucleic acids research* 2009; 37: 1-13.

14 Szklarczyk D, Gable AL, Lyon D, Junge A, Wyder S, Huerta-Cepas J *et al*. STRING v11: protein-protein association networks with increased coverage, supporting functional discovery in genome-wide experimental datasets. *Nucleic acids research* 2019; 47: D607-d613.

15 Bankhead P, Loughrey MB, Fernández JA, Dombrowski Y, McArt DG, Dunne PD *et al*. QuPath: Open source software for digital pathology image analysis. *Sci Rep* 2017; 7: 16878.

16 Irizarry RA, Hobbs B, Collin F, Beazer-Barclay YD, Antonellis KJ, Scherf U *et al*. Exploration, normalization, and summaries of high density oligonucleotide array probe level data. *Biostatistics* 2003; 4: 249-264.

17 Gautier L, Cope L, Bolstad BM, Irizarry RA. affy--analysis of Affymetrix GeneChip data at the probe level. *Bioinformatics* 2004; 20: 307-315.

18 Colaprico A, Silva TC, Olsen C, Garofano L, Cava C, Garolini D *et al*. TCGAbiolinks: an R/Bioconductor package for integrative analysis of TCGA data. *Nucleic acids research* 2016; 44: e71.

19 Mounir M, Lucchetta M, Silva TC, Olsen C, Bontempi G, Chen X *et al*. New functionalities in the TCGAbiolinks package for the study and integration of cancer data from GDC and GTEx. *PLoS Comput Biol* 2019; 15: e1006701.

20 Love MI, Huber W, Anders S. Moderated estimation of fold change and dispersion for RNA-seq data with DESeq2. *Genome Biol* 2014; 15: 550.

21 Tibshirani R. Estimating Transformations for Regression via Additivity and Variance Stabilization. *Journal of the American Statistical Association* 1988; 83: 394-405.

22 Sheffer M, Bacolod MD, Zuk O, Giardina SF, Pincas H, Barany F *et al*. Association of survival and disease progression with chromosomal instability: A genomic exploration of colorectal cancer. *Proceedings of the National Academy of Sciences* 2009; 106: 7131-7136.

23 Lu X, Parvathaneni S, Hara T, Lal A, Sharma S. Replication stress induces specific enrichment of RECQ1 at common fragile sites FRA3B and FRA16D. *Mol Cancer* 2013; 12: 29.

24 Schneider-Stock R, Ribatti D. The CAM Assay as an Alternative In Vivo Model for Drug Testing. *Handb Exp Pharmacol* 2021; 265: 303-323.

25 Muenzner JK, Kunze P, Lindner P, Polaschek S, Menke K, Eckstein M *et al*. Generation and characterization of hepatocellular carcinoma cell lines with enhanced cancer stem cell potential. *J Cell Mol Med* 2018; 22: 6238-6248.

26 Lindner P, Paul S, Eckstein M, Hampel C, Muenzner JK, Erlenbach-Wuensch K *et al*. EMT transcription factor ZEB1 alters the epigenetic landscape of colorectal cancer cells. *Cell Death Dis* 2020; 11: 147.

27 Comenge J, Sharkey J, Fragueiro O, Wilm B, Brust M, Murray P *et al*. Multimodal cell tracking from systemic administration to tumour growth by combining gold nanorods and reporter genes. *Elife* 2018; 7.

28 Stewart SA, Dykxhoorn DM, Palliser D, Mizuno H, Yu EY, An DS *et al*. Lentivirus-delivered stable gene silencing by RNAi in primary cells. *RNA* 2003; 9: 493-501.

29 Coughlan AM, Harmon C, Whelan S, O'Brien EC, O'Reilly VP, Crotty P *et al*. Myeloid Engraftment in Humanized Mice: Impact of Granulocyte-Colony Stimulating Factor Treatment and Transgenic Mouse Strain. *Stem Cells Dev* 2016; 25: 530-541.

30 Shultz LD, Lyons BL, Burzenski LM, Gott B, Chen X, Chaleff S *et al*. Human lymphoid and myeloid cell development in NOD/LtSz-scid IL2R gamma null mice engrafted with mobilized human hemopoietic stem cells. *J Immunol* 2005; 174: 6477-6489.
