# Supplementary figures and images for: ATF2 loss promotes tumor invasion in colorectal cancer cells via upregulation of cancer driver TROP2
Source: Cell Mol Life Sci. 2022 Jul 15;79(8):423. doi: 10.1007/s00018-022-04445-5 (PMC9287261; doi:10.1007/s00018-022-04445-5)

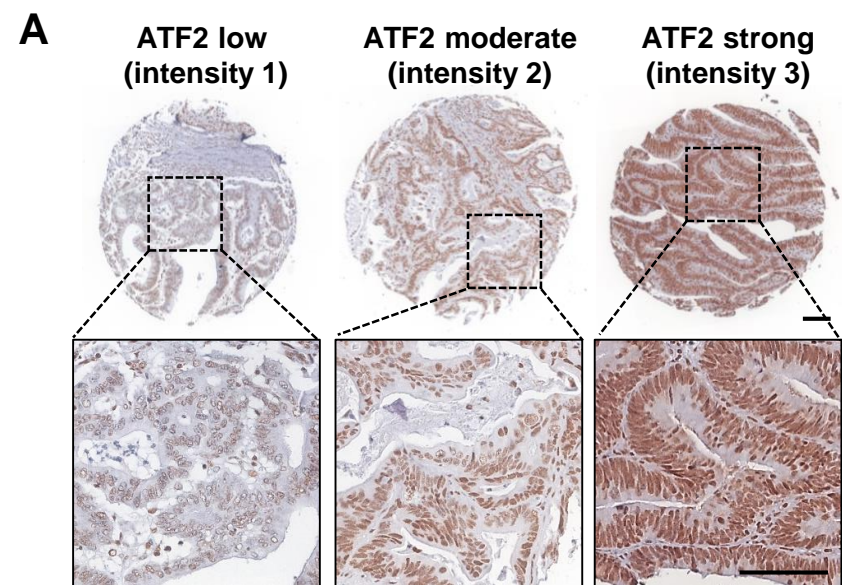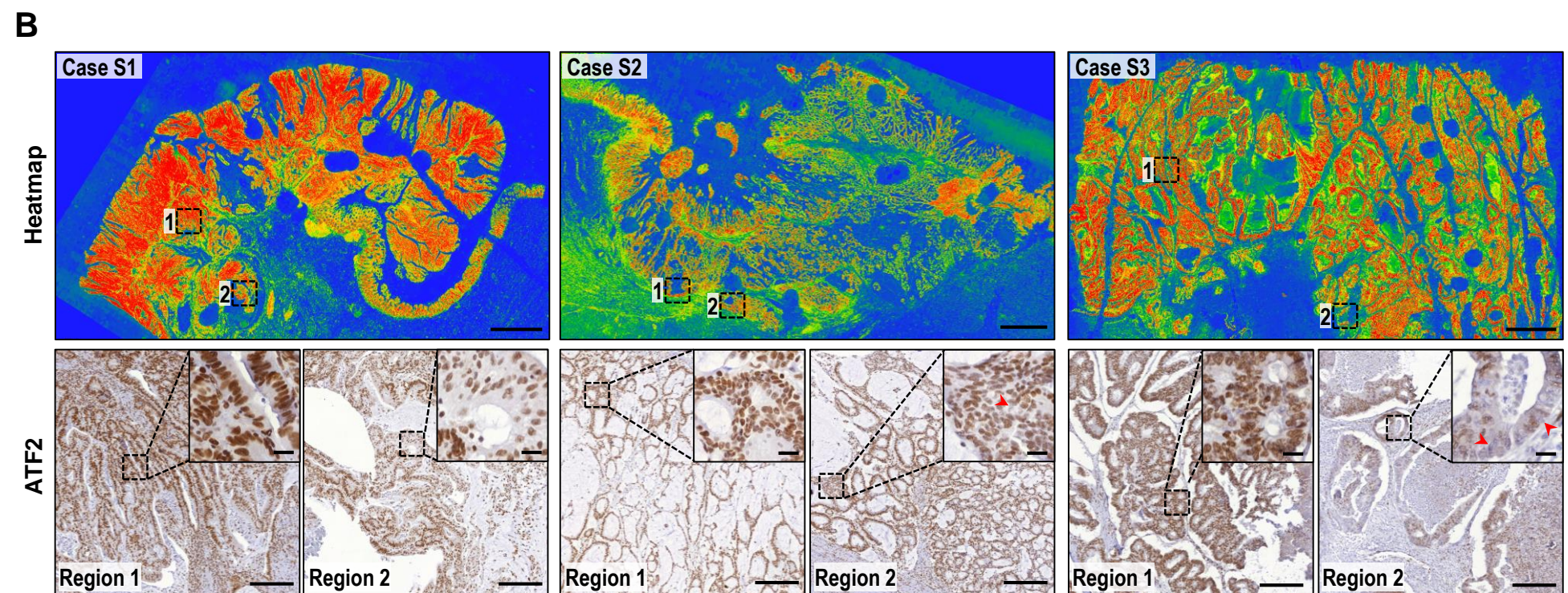

**Supplementary Figure 1**

Supplement: Supplementary file 2 — Supplementary file2 (PDF 594 KB) [file 18_2022_4445_MOESM2_ESM.pdf]

A

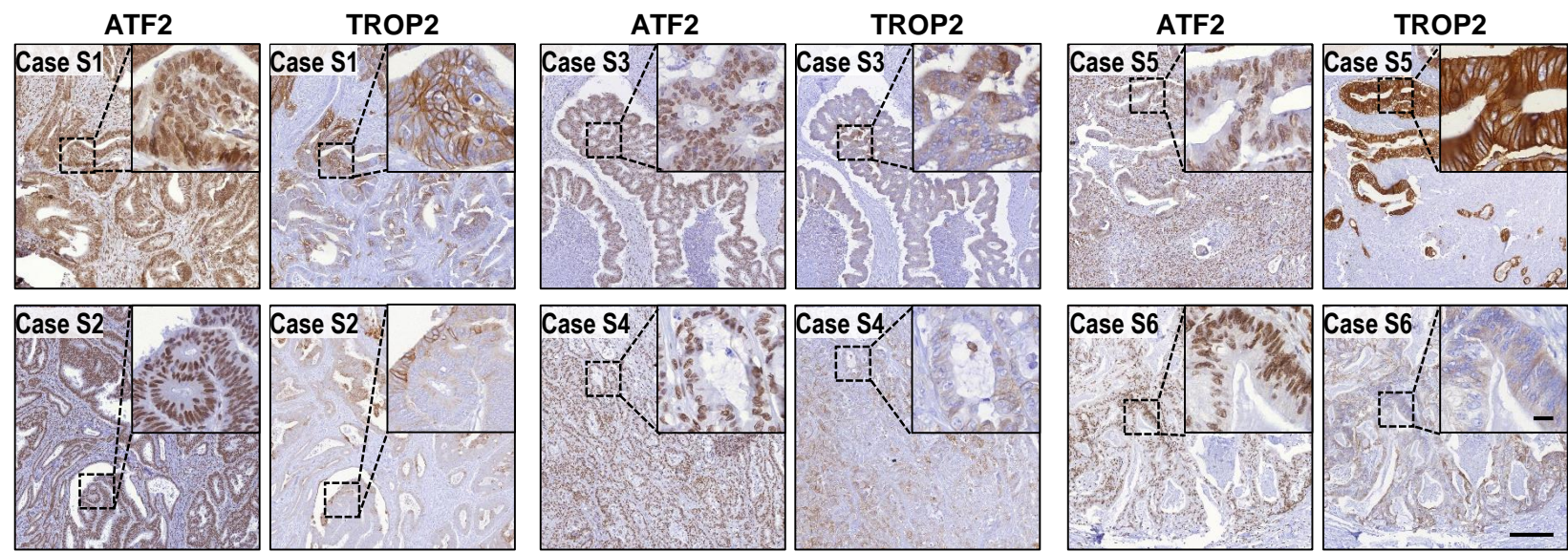

B

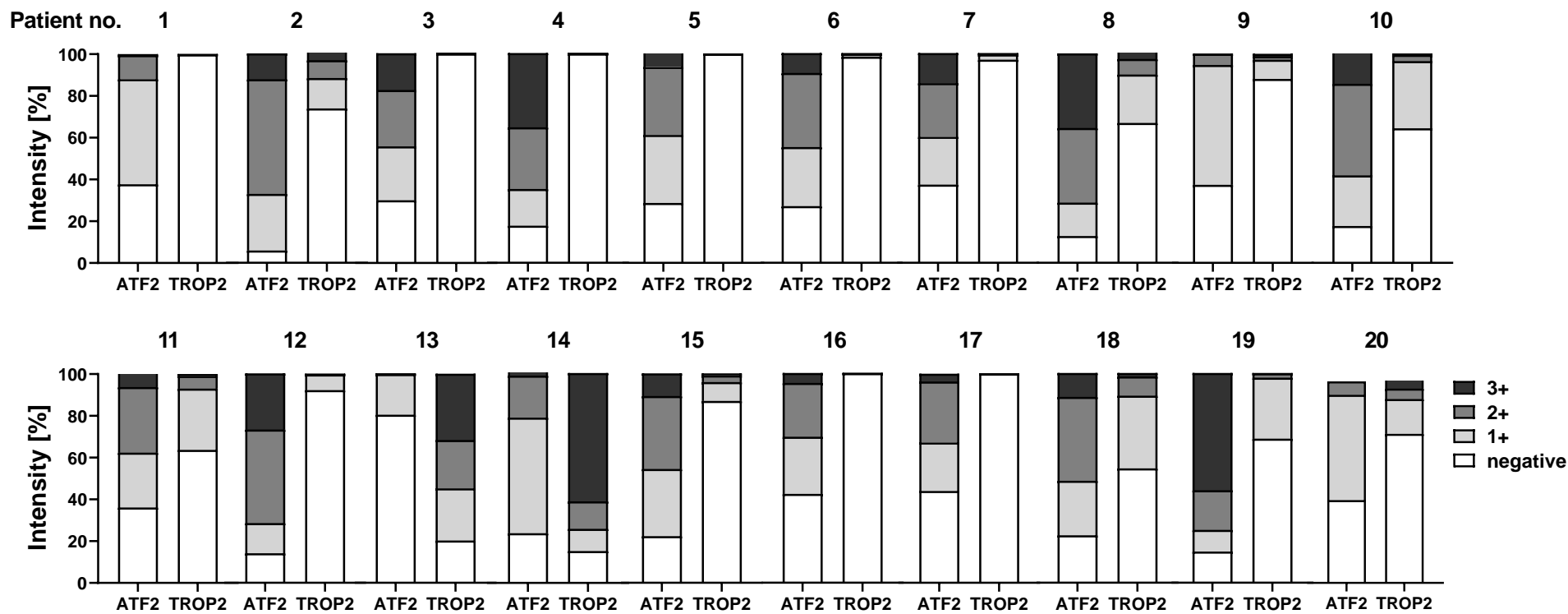

Supplementary Figure 3

Supplement: Supplementary file 4 — Supplementary file4 (PDF 497 KB) [file 18_2022_4445_MOESM4_ESM.pdf]

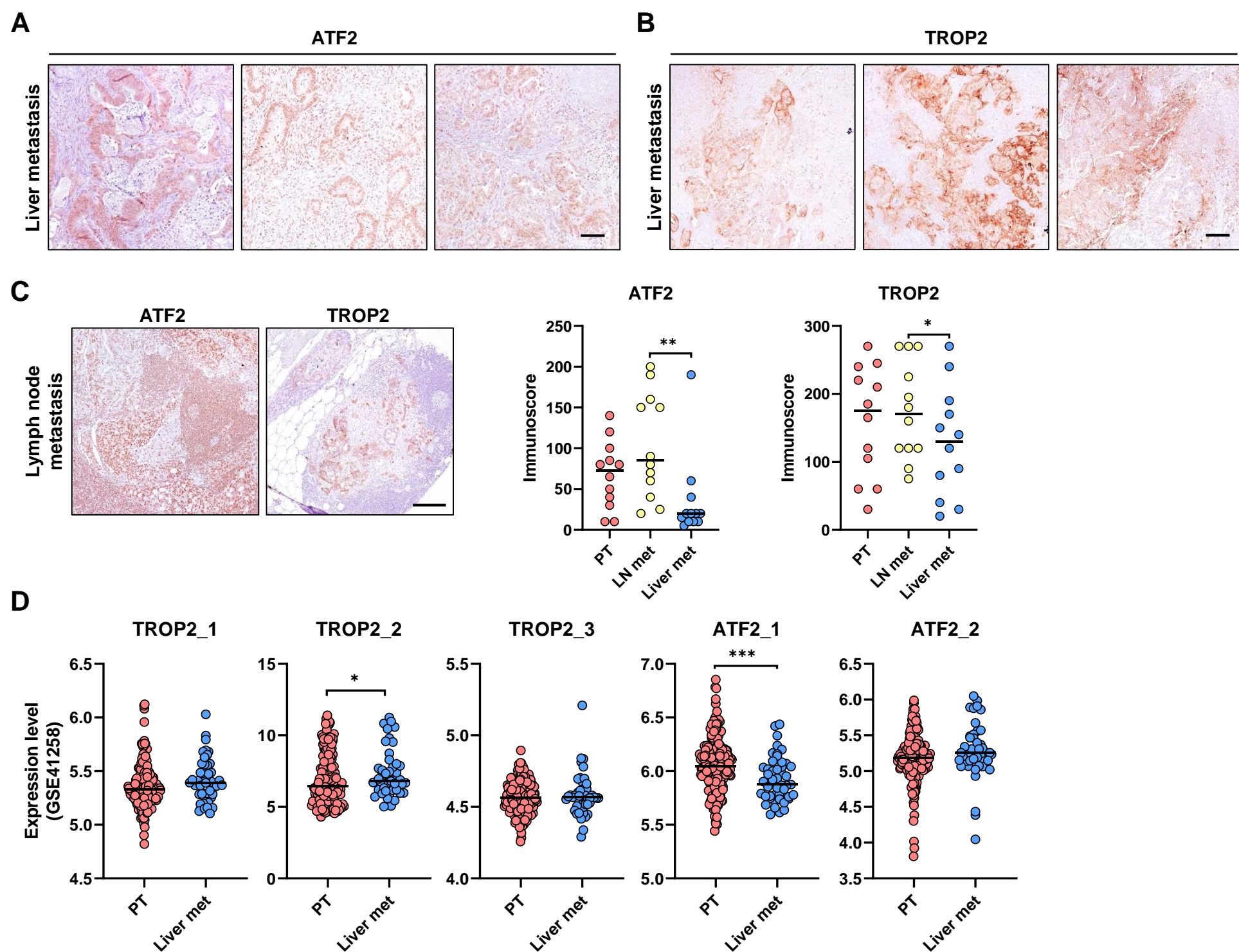

**Supplementary Figure 4**

Supplement: Supplementary file 5 — Supplementary file5 (PDF 1576 KB) [file 18_2022_4445_MOESM5_ESM.pdf]

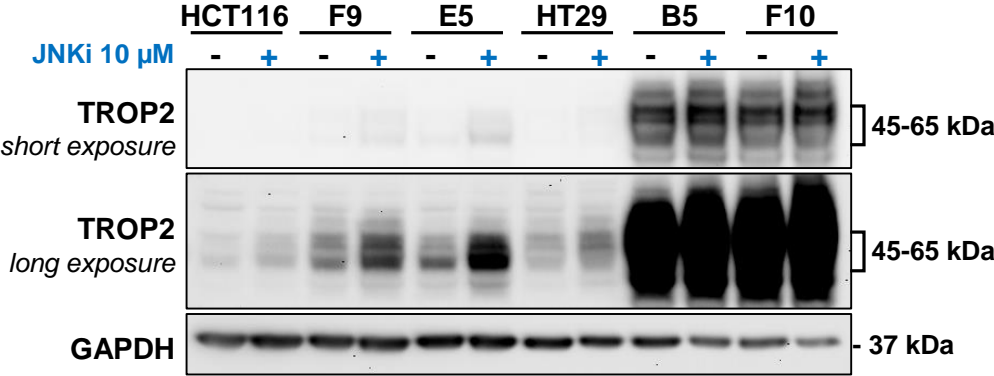

**Supplementary Figure 5**

Supplement: Supplementary file 6 — Supplementary file6 (PDF 22 KB) [file 18_2022_4445_MOESM6_ESM.pdf]

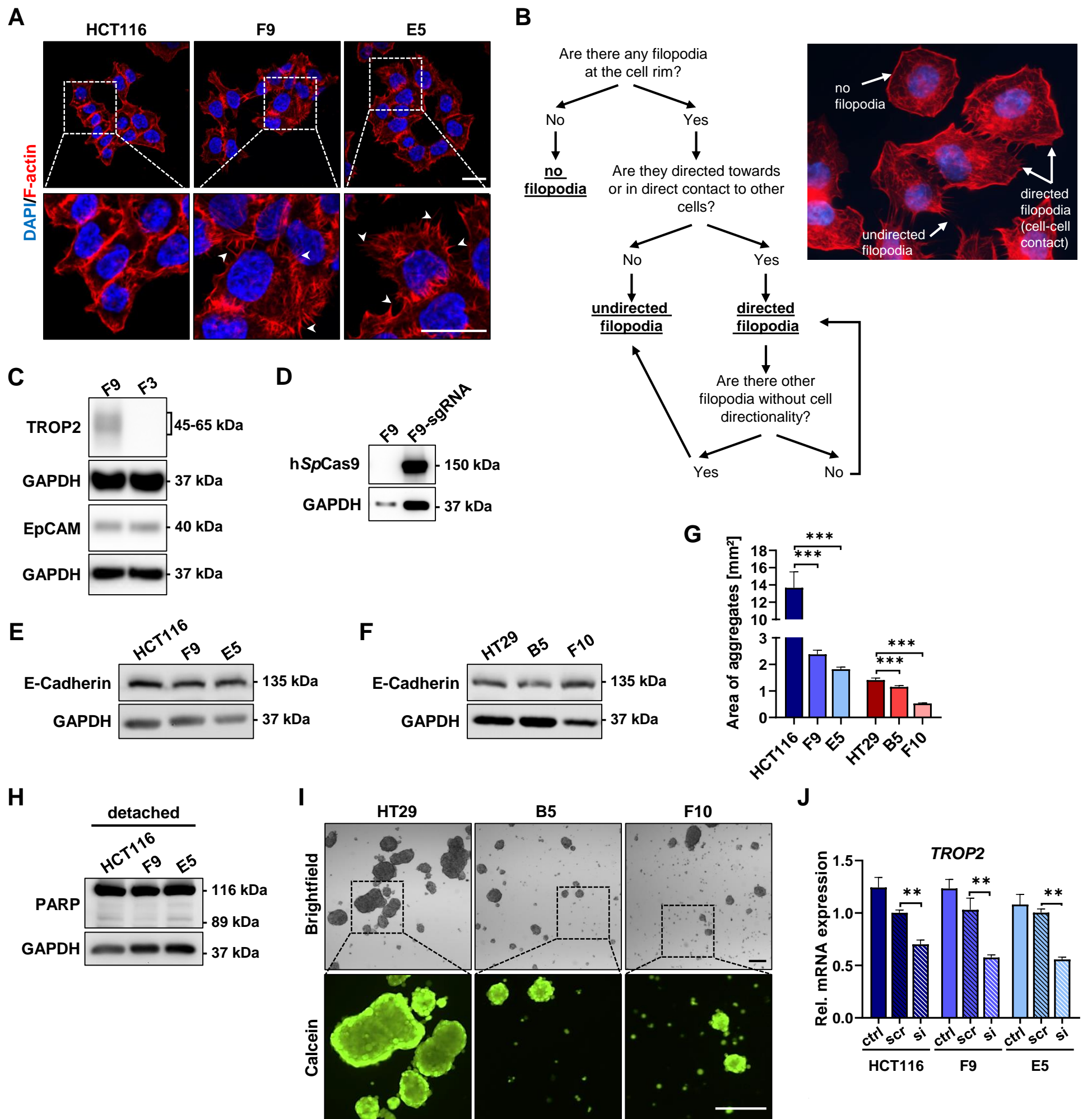

**Supplementary Figure 6**

Supplement: Supplementary file 7 — Supplementary file7 (PDF 218 KB) [file 18_2022_4445_MOESM7_ESM.pdf]

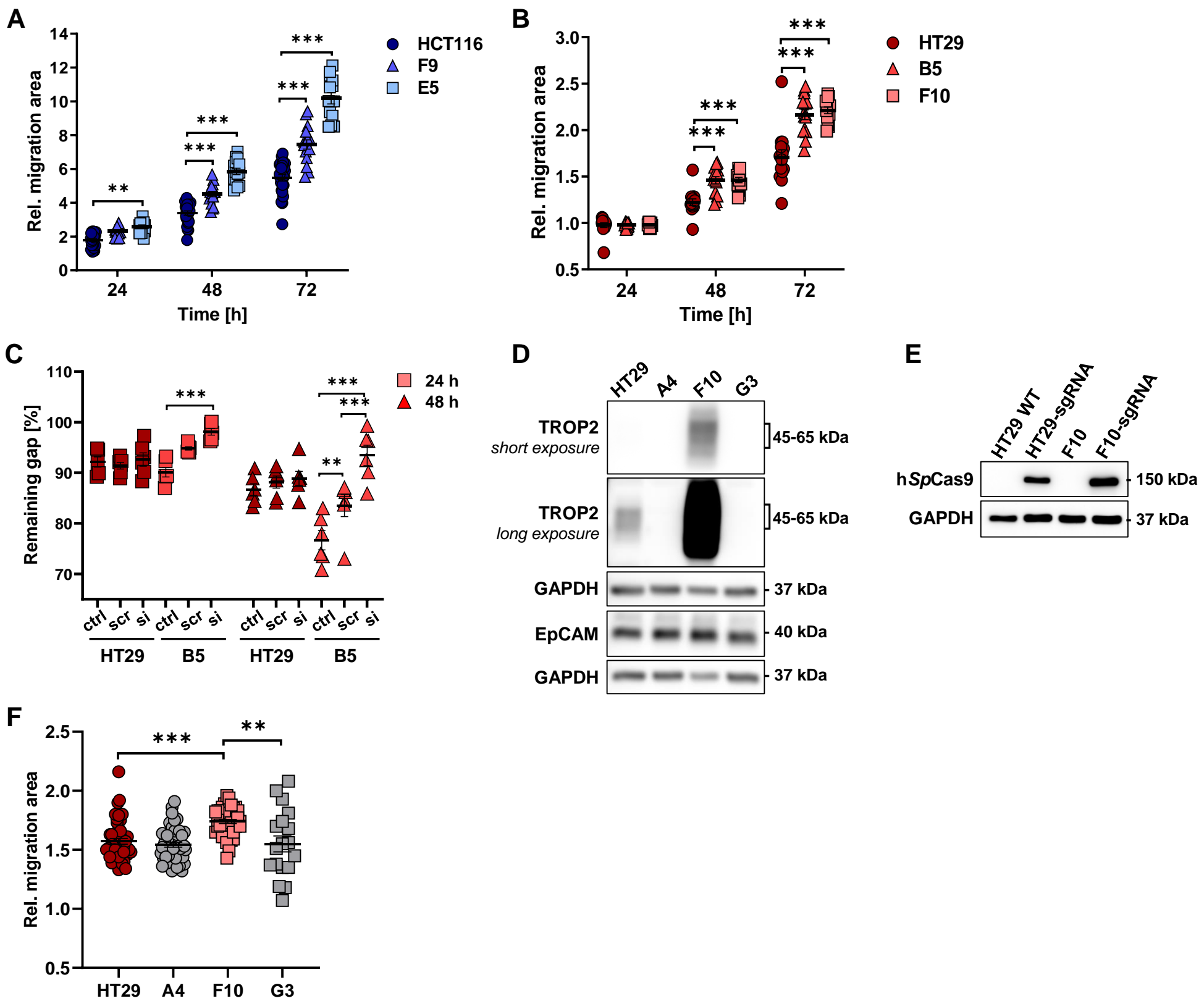

Supplementary Figure 7

Supplement: Supplementary file 8 — Supplementary file8 (PDF 102 KB) [file 18_2022_4445_MOESM8_ESM.pdf]

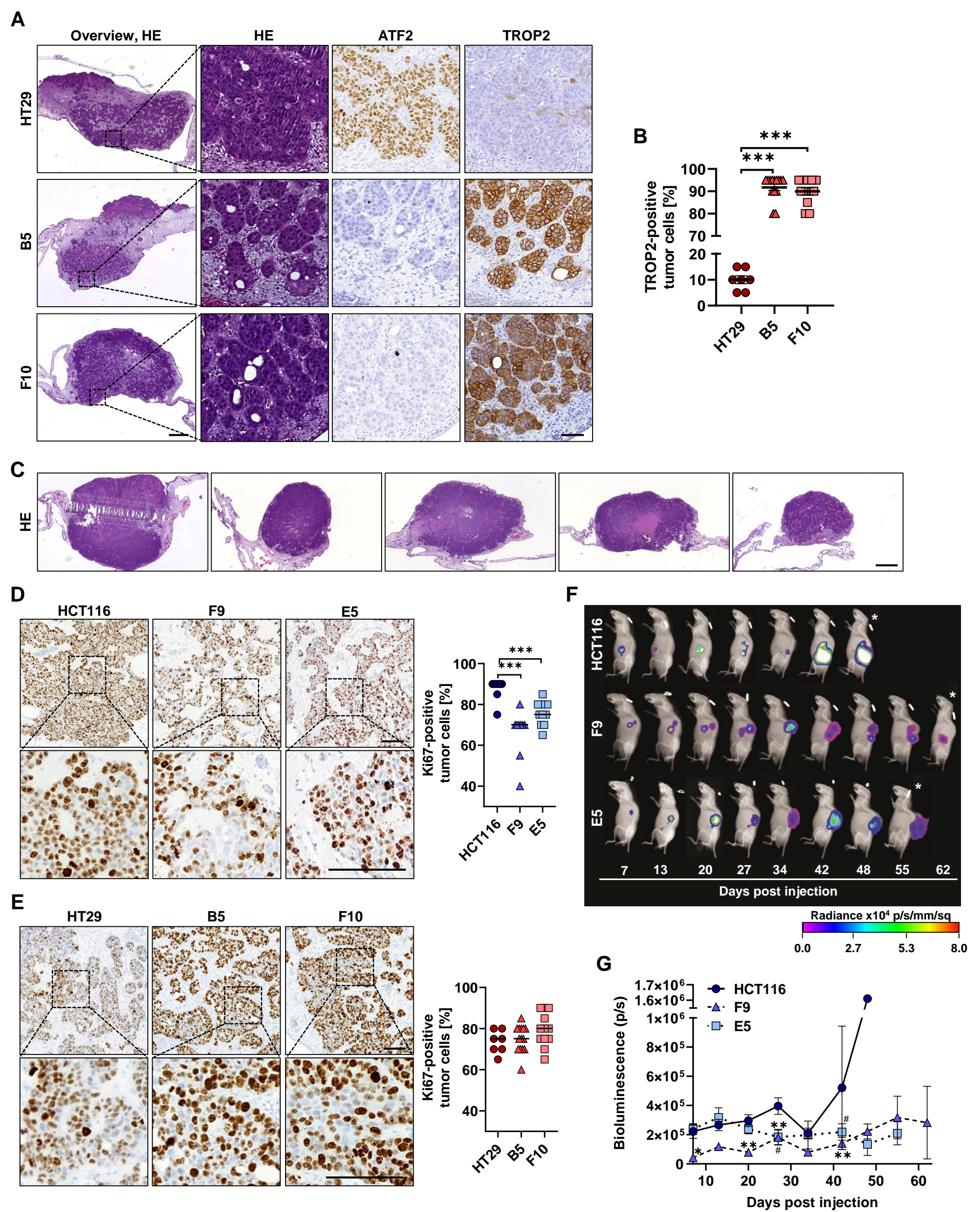

Supplementary Figure 8

Supplement: Supplementary file 9 — Supplementary file9 (PDF 656 KB) [file 18_2022_4445_MOESM9_ESM.pdf]
