# Supplementary material for: ATF2 loss promotes tumor invasion in colorectal cancer cells via upregulation of cancer driver TROP2
Source: Cell Mol Life Sci. 2022 Jul 15;79(8):423. doi: 10.1007/s00018-022-04445-5 (PMC9287261; doi:10.1007/s00018-022-04445-5)
Supplement: Supplementary file 3 — Supplementary file3 (PDF 424 KB) [file 18_2022_4445_MOESM3_ESM.pdf]

A

RefSeq	Oligo name	Oligo DNA pairs[a,b]	PAM[c]	Position	ON-target score	OFF-target score	Strand
NM_001880.4 (ATF2)	hATF2_sgRNA_2_for	3'- caccGATGACACTGTCATTACGTGC -5'	TGG	exon 5	27.43	46.75	-1
	hATF2_sgRNA_2_rev	5'- aaacGCACGTAATGACAGTGTCATC -3'					+1

[a] *Bbs*I restriction site overhang: forward cacc, reverse aaac; [b] additional G-C pair for U6 transcription; [c] PAM: protospacer adjacent motif

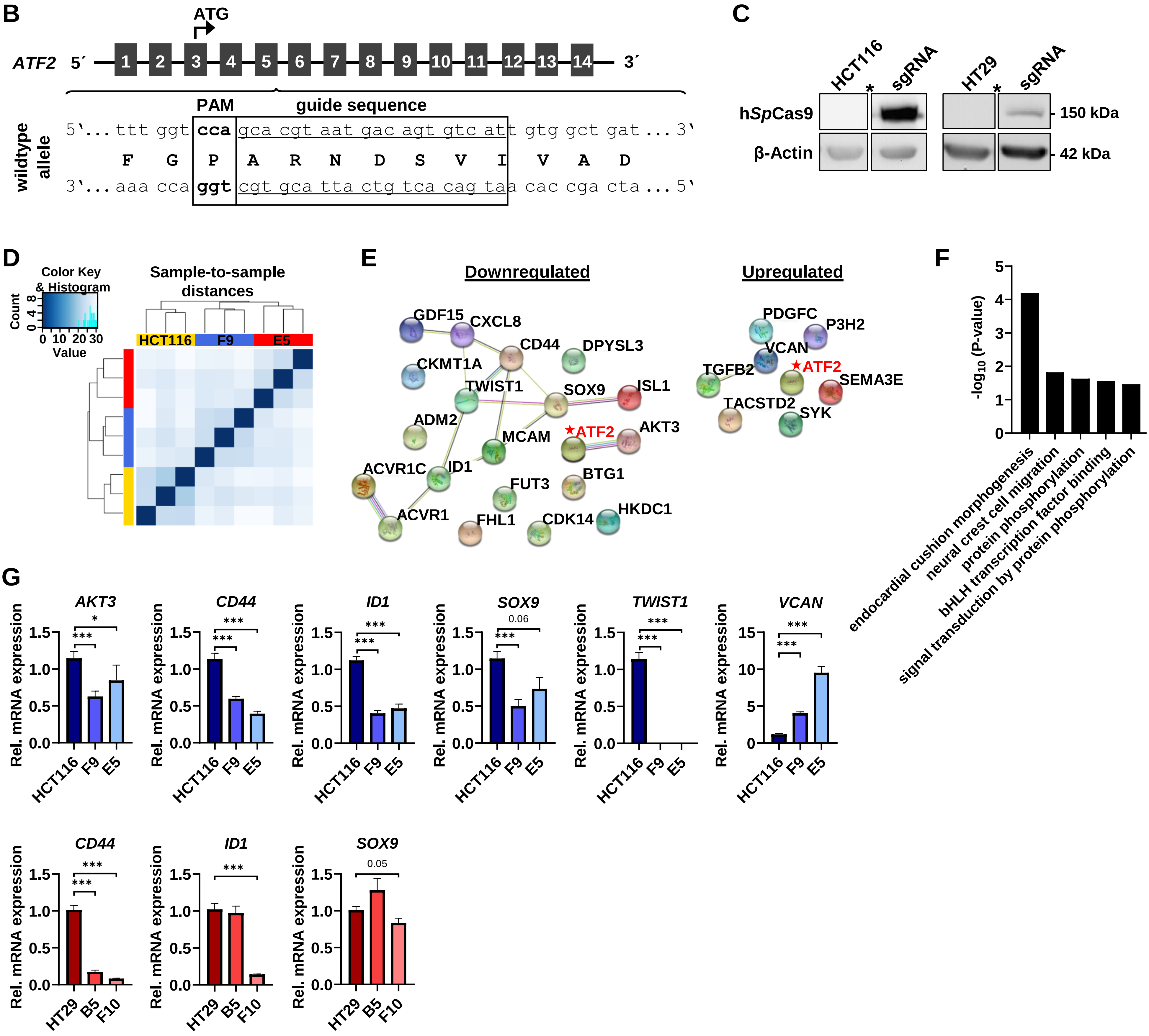

Supplementary Figure 2
